# Asymptomatic systolic dysfunction on contemporary echocardiography in anthracycline-treated long-term childhood cancer survivors: a systematic review

**DOI:** 10.1007/s11764-021-01028-4

**Published:** 2021-03-27

**Authors:** Remy Merkx, Jan M. Leerink, Esmée C. de Baat, Elizabeth A. M. Feijen, Wouter E. M. Kok, Annelies M. C. Mavinkurve-Groothuis, Jacqueline Loonen, Helena J. H. van der Pal, Louise Bellersen, Chris L. de Korte, Leontien C. M. Kremer, Elvira C. van Dalen, Livia Kapusta

**Affiliations:** 1 Department of Medical Imaging, Medical UltraSound Imaging Centre, Radboud University Medical Center, P.O. Box 9101, 6500 HB Nijmegen, The Netherlands; 2 Department of Cardiology, Amsterdam University Medical Centers, University of Amsterdam, Amsterdam, The Netherlands; 3 Princess Máxima Center for Pediatric Oncology, Utrecht, The Netherlands; 4 Department of Haematology, Radboud University Medical Center, Nijmegen, The Netherlands; 5 Department of Cardiology, Radboud University Medical Center, Nijmegen, The Netherlands; 6 Department of Paediatrics, Pediatric Cardiology Unit, Tel Aviv Sourasky Medical Centre, Sackler School of Medicine, Tel Aviv University, Tel Aviv, Israel; 7 Department of Paediatric Cardiology, Amalia Children’s Hospital, Radboud University Medical Center, Nijmegen, The Netherlands

**Keywords:** Cardiotoxicity, Systolic dysfunction, Echocardiography, Anthracyclines, Childhood cancer survivors

## Abstract

**Purpose:**

Echocardiographic surveillance for asymptomatic left ventricular systolic dysfunction (ALVSD) is advised in childhood cancer survivors (CCS), because of their risk of heart failure after anthracycline treatment. ALVSD can be assessed with different echocardiographic parameters. We systematically reviewed the prevalence and risk factors of late ALVSD, as defined by contemporary and more traditional echocardiographic parameters.

**Methods:**

We searched databases from 2001 to 2020 for studies on ≥ 100 asymptomatic 5-year CCS treated with anthracyclines, with or without radiotherapy involving the heart region. Outcomes of interest were prevalence of ALVSD—measured with volumetric methods (ejection fraction; LVEF), myocardial strain, or linear methods (fractional shortening; FS)—and its risk factors from multivariable analyses.

**Results:**

Eleven included studies represented 3840 CCS. All studies had methodological limitations. An LVEF < 50% was observed in three studies in 1–6% of CCS, and reduced global longitudinal strain (GLS) was reported in three studies in 9–30% of CCS, both after a median follow-up of 9 to 23 years. GLS was abnormal in 20–28% of subjects with normal LVEF. Abnormal FS was reported in six studies in 0.3–30% of CCS, defined with various cut-off values (< 25 to < 30%), at a median follow-up of 10 to 18 years. Across echocardiographic parameters, reported risk factors were cumulative anthracycline dose and radiotherapy involving the heart region, with no ‘safe’ dose for ALVSD.

**Conclusions:**

GLS identifies higher prevalence of ALVSD in anthracycline-treated CCS, than LVEF.

**Implications for Cancer Survivors:**

The diagnostic and prognostic value of GLS should be evaluated within large cohorts.

**Protocol registration:**

PROSPERO CRD42019126588

**Supplementary Information:**

The online version contains supplementary material available at 10.1007/s11764-021-01028-4.

## Introduction

With improved childhood cancer survival, cardiotoxicity emerges as the major non-malignant cause of late morbidity and mortality. Compared to the general population, childhood cancer survivors (CCS) have a sixfold heart failure specific mortality [1]. The cumulative incidence of symptomatic heart failure reaches 5–12%, 30 to 40 years after cancer diagnosis. Major causes are anthracyclines and radiotherapy involving the heart region [2, 3]. Hence, survivorship care focusses on early detection of left ventricular (LV) dysfunction, and guidelines recommend echocardiographic surveillance of asymptomatic CCS at least every 5 years [4].

Knowledge of asymptomatic LV systolic dysfunction (ALVSD) in CCS is important to define surveillance recommendations. A systematic review on prevalence of and risk factors for ALVSD after anthracycline treatment, with or without radiotherapy, dates from 2002 [5]. Reported systolic dysfunction varied between 0 and 38%, and denoted risk factors were cumulative anthracycline dose and follow-up duration, while age at cancer diagnosis and female sex were ambiguous risk factors. The included studies showed heterogeneity in cardiotoxic exposure and, importantly, outcome definition, and most studies had methodological limitations. The reported outcome parameters were mostly fractional shortening (FS) and rarely LV ejection fraction (LVEF), but also circumferential fibre shortening velocity and stress velocity index [5].

The introduction of strain measurement by speckle tracking, especially global longitudinal strain (GLS), has led to earlier recognition of systolic dysfunction in various cardiovascular diseases including adult cardio-oncology [6, 7]. The prevalence and risk factors for ALVSD in CCS have not been described in a systematic review addressing both strain measurements and conventional systolic function measurements.

We systematically reviewed the available literature, continuing from our last systematic review [5], on (1) the prevalence of and (2) risk factors for ALVSD, to add evidence on contemporary echocardiographic parameters such as biplane and 3D LVEF and GLS, in long-term survivors of childhood cancer treated with anthracyclines with or without radiotherapy.

## Methods

### Search strategy

We searched Medline/PubMed, EMBASE and Cochrane CENTRAL with terms for ‘anthracyclines’, ‘children’ and ‘asymptomatic systolic dysfunction’ (Online Resource 1) without language limits, from May 2001, up until April 13, 2020. We explored reference lists of included articles and narrative reviews and performed automated citation searching in Web of Science.

### Study selection

Two authors independently reviewed titles, abstracts and full-texts for potentially eligible studies. A third author solved disagreements. We included original studies evaluating at least 100 asymptomatic CCS [8], who received anthracyclines with or without radiotherapy involving the heart region. As childhood cancer types incidentally occur at later ages, 90% should be diagnosed before the age of 21 years. Echocardiographic evaluation was required at least 5 years after cancer diagnosis. As the major screening studies included some symptomatic cases, we accepted a maximum of 2.5%.

Primary outcomes were (1) prevalence of ALVSD, or (2) its risk factors derived from multivariable analysis that minimally included sex, age at diagnosis and either attained age or follow-up duration since cancer diagnosis.

We defined ALVSD according to adult [9] and pediatric [10] echocardiography guidelines: (i) a volumetric approach (e.g. reduced biplane or 3D LVEF), (ii) myocardial strain analysis (e.g. reduced GLS or global circumferential strain (GCS)), by any technique and (iii) a linear approach (e.g. reduced FS or Teichholz LVEF) although currently discouraged in adults.

Cut-off values for abnormal were adopted as stated. For strain measurements, these should be specific to the software used. Studies where outcomes were not reported separately for the defined population, cohorts with unclear (a)symptomatic status, and studies during pregnancy were excluded.

We accepted multivariable risk factor analyses to include CCS not treated with anthracyclines or with slightly shorter follow-up since diagnosis, since anthracycline dose and follow-up duration were corrected for in the analysis and no analyses were more specific. From studies reporting identical outcomes in overlapping cohorts, a combined or latest report was selected.

### Data extraction, risk of bias assessment and analysis

Abovementioned authors independently extracted data using piloted forms. Up to two written requests were sent to study authors when missing data or eligible subgroups were encountered. Authors reporting continuous values of systolic function were requested to provide the prevalence of systolic dysfunction. Risk of bias was evaluated based on previously published criteria for observational studies (Online Resource 2) [11, 12]. MetaXL 5.3 (EpiGear International) was used to calculate 95% confidence intervals of prevalences with continuity correction. Continuous values are presented as median [range], unless stated otherwise.

## Results

### Identified studies

**Fig. 1:**
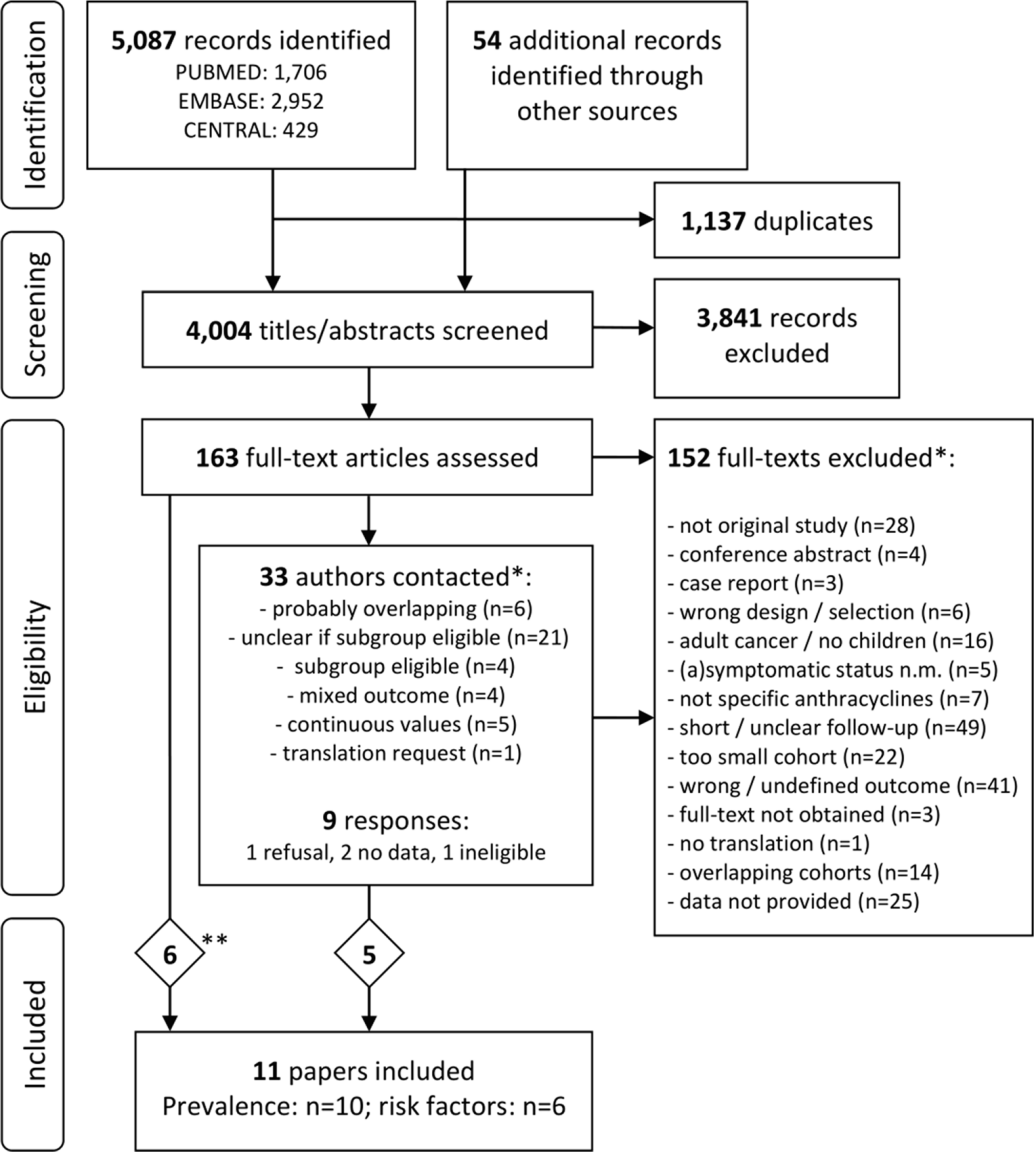
PRISMA flowchart of study selection. Flowchart describing the systematic literature search and inclusion of studies. *Multiple reasons can be given per study, references in Online Resource 3. **Although directly eligible, 2 of these 6 authors provided additional data upon request

Of the 4004 unique titles and abstracts identified, 163 were selected for full-text assessment. Additional data were received from seven studies. To address the prevalence question, ten studies were included, and for the risk factor question, six were eligible (Fig. 1, Table 1).

**Table 1:** Characteristics of included studies assessing prevalence and/or risk factors of asymptomatic left ventricular systolic dysfunction

1st Author, year country, period	Design, population	Original cohort^a^ (*n*)	Prevalence^a^								Risk factors
			Eligible (*n*) (% males)	Age at diagnosis, Years since diagnosis, Attained age (years)	Cumulative anthracycline dose (mg/m^2^)	Heart RT (*n* (%))	Current CHF (*n*)	Dexrazoxane (*n*)	Outcome definition	Prevalence (*n* (%; 95% CI)	Eligible
Slieker, 2019^b^ Canada, n.m., [13]	*n* = 546 Cross-sectional, Last ANT dose + ≥ 3 years. Attained age < 18 years No SCT, CHD or familial CMP	n.m.	467 (54)	3.4 [0.1–13.2] 9.2 [5.0–17.2] 14.1 [5.2–18.8]	166 [18–699] (Cardiotoxic doxorubicin equivalence)	49 (10)	0	17	Biplane LVEF < 50% GLS *Z*-score < − 2 (single-view, EchoPac software)	2/338 (0.6; 0–1.8) 39/435 (9; 6.4– 11.8)	Yes
Li, 2019^c^ China, n.m., [14]	*n* = 103 Prospective, cross-sectional, ANT, treatment + ≥ 5 years, attained age ≥ 15 years	n.m.	103 (55)	8.2 ± 5.0^d^ 15.2 ± 5.8 25.0 ± 5.8	220 [60–675] (Conversion factor n.m.)	5 (5)	0	n.m.	FS < 27%	1/103 (1.0; 0–4.1)	No
Armenian, 2018^b^ California, USA, 2014–2017 [15]	*n* = 221 cross-sectional, ANT, diagnosis + ≥ 2 years	n.m.	193 (52)	11.4 [< 1–22]^e^ 15.8 [5.1–44.8] 26.1 [13.0–59.9]	235 [25–642] (Haematotoxic doxorubicin equivalence)	30 (16)	1	n.m.	Biplane LVEF < 50%	11/193 (5.7; 3– 9)	No
Pourier, 2017^c^ Netherlands, 2006–2010, [16]	*n* = 340 Retrospective cross-sectional, ANT, diagnosis + ≥ 5 years, asymptomatic, no CHD	n.m.	340 (54)	5.9 [0–17.5] 13.7 [4.9–32.0] 21.3 [6.0–43.0]	180 [30–600] (Doxorubicin + daunorubicin)	49 (14)	0	n.m.	FS < 27% Teich EF < 50%	1/340 (0.3; 0–1.3) 1/340 (0.3; 0–1.3)	No
Christiansen, 2016 Norway, 2007–2011 [17]	*n* = 231 Cross-sectional, ALL/lymphoma, diagnosis + ≥ 5 years, attained age ≥ 18 years	n.m.	231^f^ (51)^f^	9.3 ± 5.1^f^ 21.9 ± 8.0^f^ 31.1 ± 7.8^f^	150 [40–485]^f^(Conversion factor n.m.)	52 (23)^f^ 40 Gy^f^	n.m.^f^	n.m.^f^	Biplane LVEF < 50% GLS > controls -1.96SD EchoPac software	Not eligible^f^	Yes
Armstrong, 2015 Tennessee, USA, n.m. [18]	*n* = 1807 Prospective cross-sectional, ANT or RT, diagnosis + ≥ 10 years, attained age ≥ 18 years	n.m.	1514 (52)	n.m. [0–>19]^e^ 22.6 [10.4–48.3] 31 [18–65]	n.m. [up to > 600] (Conversion factor n.m.)	464 (31)	17	n.m.	3D LVEF < 50% GLS > age/sex norm GCS > age/sex norm (EchoPac software)	n.m./n.m. (5.8; n.m.) n.m./n.m. (30; n.m.) n.m./n.m. (23; n.m.)	Yes
	normal LVEF only		n.m.	n.m.	n.m.	n.m.	n.m.	n.m.	GLS > age/sex norm	n.m./n.m. (28; n.m.)	
Mavinkurve-Groothuis, 2010^c^ Netherlands, 2006–2008 [19]	*n* = 109 Prospective cross-sectional, ANT, diagnosis + ≥ 5 years, no CHF/CVD/CKD	n.m.	109 (57)	4.8 [.03–16.9] 13.2 [5.0–29.2] 20 [5.6–37.4]	180 [50–600] (Doxorubicin + daunorubicin)	7 (6.3)	0	n.m.	GLS > age/sex norm GRS < age/sex norm GCS > age/sex norm (single view, EchoPac software)	22/92 (24; 16 - 33) 4/89 (4.5; 1–10) 35/82 (43; 32–54)	No
	normal LVEF only		49 (57)	5.3 [.03–16.8] 10.8 [5.0–26.2] 16.8 [5.6–34.4]	180 [50–450]	4 (8.2)	0	n.m.	GLS > age/sex norm GRS < age/sex norm GCS > age/sex norm	9/45 (20; 9.4–33) 2/43 (4.7; 1–14) 10/36 (28; 14– 44)	
van der Pal, 2010^b^ Netherlands, 1996–2004, [20]	*n* = 525 Prospective cross-sectional, ANT/RT/high dose cyclo-/ifosfamide, diagnosis + ≥ 5 years, attained age ≥ 18 years	n.m.	361 (54)	9.7 [0.1–17.8] 13.3 [5.1–28.8] 21.7 [18–42.1]	250 [33–720] (All anthracyclines added up)	58 (16)	0 (7^g^)	n.m.	FS < 30%	107/355 (30; 25 –35)	Yes
Hudson 2007^b^ Tennessee, USA, n.m., [21]	*n* = 223 Prospective cross-sectional, no CHD/CHF/chronic illness/trisomy 21/ anaemia	n.m.	217 (51)	5.5 [0–23.6]^e^ 10.2 [5.5–28.0] *16.9 [7.5–38.1]*	202 [25–510] (Conversion factor n.m.)	60 (28)	0 (2^g^)	n.m.	FS < 28%	32/213 (15; 11 – 20)	Yes
Pein, 2004 France, n.m, [22]	*n* = 205 Cross-sectional, ANT diagnosis + ≥ 15 years	416	205 (58)	5.7 [0–21]^h^ 18 [15+]^h^ n.m. [n.m.]	333 [40–600]^g^ (Conversion factor n.m.)	106 (52) 7.7Gy	0	n.m.	FS < 25% Teich EF < 50%	13/205 (6.3; 3.3–10.1) 17/205 (8.3; 4.9–12.5)	Yes
von der Weid, 2001 Switzerland 1994–1996, [23]	*n* = 150 Prospective, cross-sectional, ALL, no BMT diagnosis + ≥ 5 years therapy + ≥ 2 years	n.m.	140 (n.m.)	n.m. [n.m.] n.m. [5+] n.m. [n.m.]	n.m. [n.m.]	n.m.	0	n.m.	FS < 30%	2/140 (1.4; 0–4.3)	No

Numbers are medians [range] unless stated otherwise. Only two studies reported early cancer therapy related cardiotoxicity and two reported median RT dose. None reported mitoxantrone dose or infusion duration.

^a^Data shown for symptomatic survivors, ≥ 5 years from diagnosis, treated with anthracyclines

Authors ^b^ provided subgroup data; ^c^converted continuous values into prevalence data

^d^Mean ± SD, follow-up from end of therapy

^e≥^90% were diagnosed before age 21 years

^f^Data presented for entire cohort (*n* = 231) including non-anthracycline treated CCS, study not included for prevalence estimation ^g^Transient CHF during cancer therapy

^h^Mean (range)

*ALL*, acute lymphoblastic leukaemia; *ANT*, anthracyclines; *BMT*, bone marrow transplant; *CHD* , congenital heart disease; *CHF*, congestive heart failure; *CKD*, chronic kidney disease; *CMP*, cardiomyopathy; *CVD*, cardiovascular disease; *GCS*, global circumferential strain; *GLS*, global longitudinal strain; *GRS*, global radial strain; *FS*, fractional shortening; *Heart RT*, radiotherapy involving the heart region, as defined by individual study; *LVEF*, left ventricular ejection fraction; *SCT*, stem cell transplant; *Teich EF*, left ventricular ejection fraction according to Teichholz formula; *n.m*., not mentioned

Three studies (2174 CCS) used a volumetric approach (biplane LVEF *n* = 660, 3D LVEF *n* = 1514) to quantify ALVSD [13, 15, 18]. Myocardial strain was reported in four studies (*n* = 2281). One of these studies used vendor specific normative values for GLS and GCS [18], another study in a pediatric cohort defined abnormal GLS (apical 4-chamber view) as vendor specific z-score < − 2 [13]. A third study reported apical 4-chamber GLS and mid-ventricular GCS as continuous values, and compared to normative values for adults [24] and children [25] upon our request [19]. The fourth study reporting GLS was only eligible for its risk factor analysis [17]. Two of these studies compared myocardial strain to LVEF [18, 19]. A linear approach was reported in six studies (FS *n* = 1366, Teichholz LVEF *n* = 557). Two studies reported continuous values and provided prevalences according to their local cut-off values upon request [14, 16, 20–23].

Median follow-up from cancer diagnosis until echocardiographic examination varied between the studies from 9 to 23 years, as did the proportion of survivors who received radiotherapy involving the heart region (5–52%). Median cumulative anthracycline dose ranged from 166 to 333 mg/m^2^, but studies used different dose-equivalence ratios.

### Risk of bias assessment

**Fig. 2:**
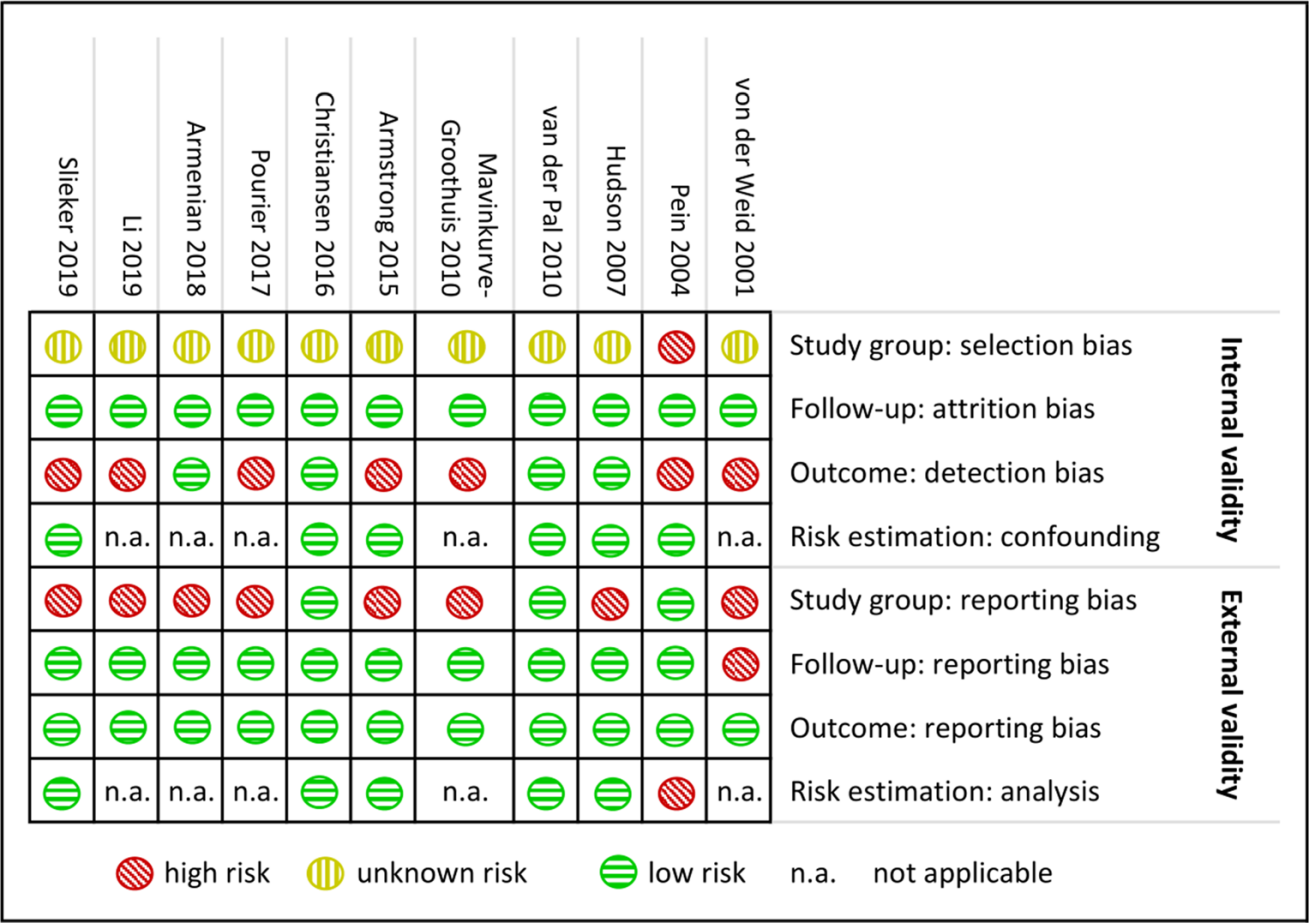
Risk of bias summary per study. The risk of bias per study is indicated for each domain. Assessment criteria are shown in Online Resource 2. Green = low risk; yellow = unknown risk; red = high risk; n.a, is not applicable

Figure 2 depicts the risk of bias assessment. Ninety-one percent of the studies did not report original cohort sizes and thus risk of selection bias remained unclear; in 9% the risk was high. Four studies (36%) reported blinded outcome assessment; the remainder carried a high risk of detection bias. All six studies assessing risk factors in a multivariable analysis had low risk of confounding. The risk of study group reporting bias was high in 73%. Not all studies reported median cumulative anthracycline dose; only three studies summarized radiotherapy doses involving the heart region, and only one reported additional chemotherapeutic agents. Follow-up duration was summarized by 91% of the studies, and all studies provided their outcome definition. Risk estimation was not adequate in 17% of the 6 studies assessing risk factors. The few studies for each outcome prevented formal testing for publication bias. However, as we searched all major databases and most studies were not industry funded, we judge the risk of publication bias ‘low’.

### Prevalence of asymptomatic systolic dysfunction

#### Volumetric methods

**Fig. 3:**
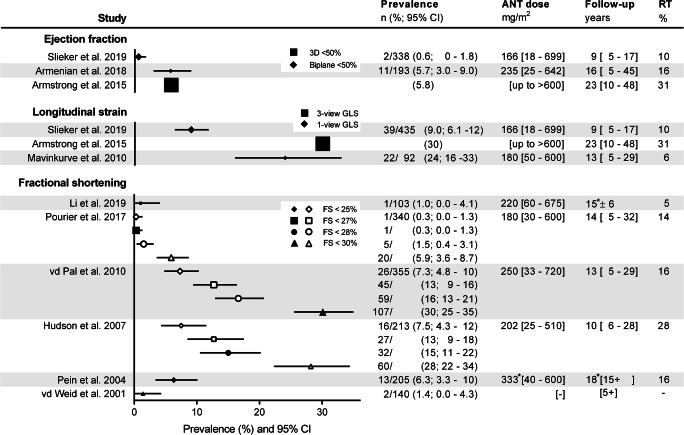
Prevalence of asymptomatic left ventricular systolic dysfunction in childhood cancer survivors. Prevalence is depicted for different echocardiographic parameters and cut-off points in the included studies. *Mean ± SD. Closed symbols depict the original cut-offs from the studies, open symbols were extracted from additional data provided by authors. Symbol size depicts sample size. Continuous values are median [range]. ANT = anthracycline, CI = confidence interval, FS = fractional shortening, GLS = global longitudinal strain, RT = radiotherapy on the heart region

Three studies, all defining an abnormal biplane or 3D LVEF < 50%, reported a prevalence of 1–6% (Fig. 3). The prevalence was lowest in the study with the shortest median follow-up duration (9 years, versus 16 and 23 years). Anthracycline doses varied. Not all studies reported a median dose. The proportion that received radiotherapy on the heart region varied from 10 to 31%[13, 15, 18]. This observed clinical heterogeneity prevented pooling of results.

#### Myocardial strain analysis

Three studies assessing myocardial strain reported abnormal GLS, according to vendor-, age- and sex-specific cut-off values, in 9–30% (Fig. 3). The lowest prevalence was again seen in the study with the shortest median follow-up duration (9 years, versus 13 and 23 years). Anthracycline doses varied. Not all studies reported a median dose. The proportion that received radiotherapy on the heart region varied from 6 to 31% [13, 18, 19]. We did again not pool results from these heterogeneous cohorts. Two studies reported GLS in subjects with normal LVEF, which was abnormal in 20–28% (Table 1) [18, 19].

Of note, in one study, only 20% of survivors with abnormal LVEF also had abnormal GLS[19]. Two studies reported higher[19], or lower[18] prevalence of abnormal GCS, compared to the prevalence of abnormal GLS.

#### Linear methods

Prevalence of abnormal FS and Teichholz LVEF varied between the six studies from 0.3 to 30%, using different definitions of abnormal FS (< 25 to < 30%; Fig. 3). As median follow-up duration (10 to 18 years) and anthracycline dose (180–250 mg/m^2^) and proportion that received radiotherapy involving the heart region (5–28%) varied widely, we did not pool results. No direct comparisons between FS and other ALVSD parameters were found in these studies.

### Risk factors

Five out of six studies that reported multivariable risk factor analyses on either dichotomous or continuous outcomes (Table 2) agreed on the incremental risk of ALVSD with increasing cumulative anthracycline dose [17, 18, 20–22]. For abnormal LVEF and FS, the risk ratios increased with higher dose categories. However, in the study assessing GLS, the risk ratios showed a more stable elevation throughout dose categories, compared to LVEF [18]. Furthermore, GLS as a continuous outcome variable was not associated with anthracycline dose [13]. Either radiation exposure or dose to the heart region were identified as risk factors by three out of four studies that assessed radiotherapy, and across all systolic function parameters [17, 18, 20]. Younger age at diagnosis and shorter follow-up duration were associated with abnormal FS in one of the three studies that analysed these variables [20]. Only one study found a sex association with, discrepantly, more males with an LVEF < 50%, but more females with abnormal GLS (sex-specific normative values). The same study analysed traditional cardiovascular risk factors and found hypertension associated with an abnormal LVEF and all components of the metabolic syndrome and attained age to be associated with an abnormal GLS [18].

**Table 2:** Reported risk factors for asymptomatic left ventricular systolic dysfunction

1st Author, year	Population	Outcome definition; (% abnormal)	Tested risk factors (reference category)	Tested categories (effect size; 95% confidence interval)	Model comments
Slieker, 2019 [13]	*n* = 546 Last anthracycline dose + ≥ 3 years. Attained age < 18 years No stem cell transplant, congenital heart disease or familial cardiomyopathy	GLS *Z*-score (continuous)	Attained age, years	**β** ***−*0.086; −0.140–** ***−*0.031**	Includes 14% survivors 3-5 years since diagnosis
			Age at diagnosis, years	n.m.^a^	
			Female sex	n.m.^a^	
			Body surface area per 0.1 m^2^ increment	**β** ***−*0.065;** ***−*0.118– −0.013**	
			Years since last anthracycline dose	n.m.^a^	
			Heart RT exposure	n.m.^a^	
			Anthracycline dose per 50 mg/m^2^ increment	n.m.^a^	
			Dexrazoxane therapy	n.m.^a^	
Christiansen, 2016 [17]	*n* = 231 Acute lymphoblastic leukaemia/lymphoma, diagnosis + ≥ 5 years, attained age ≥ 18 years	GLS > − 18.3% (female) > − 17.2% (male) (32%)	Age at diagnosis	OR 0.96; 0.90–1.03	23% had no anthracycline exposure
			Attained age	OR 1.02; 0.98–1.06	
			Heart RT exposure	**OR 5.2; 2.2–12**	
			Anthracycline dose (< 300 mg/m^2^)	***>*300 (OR 4.8; 1.7–14)**	
Armstrong, 2015 [18]	*n* = 1807 any cancer, anthracycline or Heart RT, diagnosis + ≥ 10 years, attained age ≥ 18 years	3D LVEF < 50% (5.8%)	Ethnicity (non-Hispanic white)	Other (RR 1.53; 0.93–2.52)	17% had no anthracycline exposure
			Female sex	**RR 0.54; 0.36–0.83**	
			Age at diagnosis (≥ 15 years)	0*–*4 (RR 0.66; 0.35–1.27), 5–9 (RR 0.67; 0.36–1.25), 10–14 (RR 1.02; 0.59–1.76)	
			Attained age (18–30 years)	31–40 (RR 1.38; 0.81– −2.35), > 40 (RR 0.98; 0.52–1.84)	
			Heart RT dose (0 Gy)	1-19 (RR 1.24; 0.70–2.22), **20–29 (RR 1.86; 1.00–3.45), ≥ 30 (RR 7.99; 3.88–16.48)**	
			Anthracycline dose (0 mg/m^2^)	1–100 (RR 1.74; 0.66–4.61), **101-200 (RR 2.80;** **1.24–6.31), 201-300 (RR 3.80; 1.59–9.10), 301-400 (RR 4.76; 2.16 – 10.50), > 400 (RR 7.71; 3.04 – 19.57)**	
			Metabolic syndrome (≥ 3 of the following)	RR 1.07; 0.74–1.53	
			Waist circumference > 102 (male) > 88 cm (female)	RR 1.34; 0.99-1.82	
			Triglycerides > 150 mg/dl	RR 1.01; 0.70–1.44	
			HDL < 40 (male) < 50 mg/dl (female)	RR 1.01; 0.74–1.38	
			Blood pressure ≥ 130/ and/or /85 mmHg or treated	**RR 1.44; 1.22–1.70**	
			Fasting glucose > 100 mg/dl or diabetes treatment	RR 1.02; 0.75–1.39	
		GLS > age/sex norm (31.8%)	Ethnicity (non-Hispanic white)	**Other (RR 1.22; 1.03–1.46)**	
			Female sex	**RR 1.55; 1.34–1.79**	
			Age at diagnosis (≥ 15 years)	0–4 (RR 1.02; 0.82–1.27), 5–9 (RR 0.92; 0.74 – 1.15), 10–14 (RR 1.02; 0.83–1.24)	
			Attained age (18–30 years)	**31–40 (RR 1.25; 1.05–1.48), > 40 (RR 1.49; 1.20–1.85)**	
			Heart RT dose (0 Gy)	**1–19 (RR 1.38; 1.14–1.66), 20–29 (RR 1.65; 1.31– 2.08), ≥ 30 (RR 2.39; 1.79–3.18)**	
			Anthracycline dose (0 mg/m^2^)	**1–100 (RR 1.38; 1.05–1.82)**, 101–200 (RR 1.16; 0.89–1.50), 201–300 (RR 1.06; 0.78–1.45), **301–400 (RR 1.72; 1.31–2.26), > 400 (RR 1.73; 1.19–2.50)**	
			Metabolic syndrome (≥ 3 of the following)	**RR 1.94; 1.66–2.28**	
			Waist circumference > 102 (male) > 88 cm (female)	**RR 1.73; 1.48–2.01**	
			Triglycerides > 150 mg/dl	**RR 1.65; 1.40–1.95**	
			HDL < 40 (male) < 50 mg/dl (female)	**RR 1.40; 1.23–1.59**	
			Blood pressure ≥ 130/and/or /85 mmHg or treated	**RR 1.48; 1.33–1.65**	
			Fasting glucose > 100 mg/dl or diabetes treatment	**RR 1.37; 1.19–1.59**	
		GCS > age/sex norm (23.1%)	Ethnicity (non-Hispanic white)	Other (RR 0.84; 0.64–1.09)	
			Female sex	RR 1.01; 0.84–1.21	
			Age at diagnosis (≥ 15 years)	0–4 (RR 1.24; 0.92–1.67), 5–9 (RR 1.01; 0.74–1.38), 10–14 (RR 1.11; 0.84–1.48)	
			Attained age (18–30 years)	31–40 (RR 0.85; 0.69–1.06), > 40 (RR 0.98; 0.73–1.33)	
			Heart RT dose (0 Gy)	1–19 (RR 0.86; 0.66–1.11), 20–29 (RR 1.14; 0.83–1.57), **≥ 30 (RR 1.64; 1.05–2.56)**	
			Anthracycline dose (0 mg/m^2^)	1–100 (RR 0.99; 0.66–1.48), 101–200 (RR 1.24; 0.86–1.79), 201–300 (RR 1.36; 0.90 – 2.04), **301–400 (RR 1.61; 1.08–2.40), >** 400 (RR 1.34; 0.78–2.31)	
			Metabolic syndrome (≥ 3 of the following)	RR 1.02; 0.84–1.24	
			Waist circumference > 102 (male) > 88 cm (female)	RR 1.10; 0.92–1.32	
			Triglycerides > 150 mg/dl	RR 1.01; 0.82–1.13	
			HDL < 40 (male) < 50 mg/dl (female)	RR 0.92; 0.78–1.08	
			Blood pressure ≥ 130/ and/or /85 mmHg or treated	RR 1.04; 0.92–1.18	
			Fasting glucose > 100 mg/dl or diabetes treatment	RR 1.06; 0.89–1.25	
van der Pal, 2010 [20]	*n* = 525 any cancer, anthracycline/RT/high dose cyclo-/ifosfamide, diagnosis + ≥5 years, attained age ≥ 18 years	FS (continuous)	Male sex	β 0.77 (**−**0.27–1.80*)*	31% had no anthracycline exposure
			Age at diagnosis (>15 years)	**0–5 (β −3.55; −5.80– −1.30) > 5–10 (β** **−1.95; −4.03–0.12), > 10–15 (β −1.32; −3.21–0.58)**^**b**^	
			Time since diagnosis (5-10 years)	**10–15 (β 0.41; −1.25–2.08), 15–20 (β 1.71; −0.07–3.50), 20–25 (β 2.07; −0.08–4.22), > 25 years (β 4.86; 2.28–7.43)**^**b**^	
			Vincristine exposure	β −1.30; −2.88–0.27	
			Anthracycline dose (0–150 mg/m^2^)	**151–300 (β −1.93; −3.71− −0.15), 301–450 (β −4.24; -6.32− −2.16), > 450 (β −5.38; −7.98− −2.79)**^**b**^	
			Cyclophosphamide (≤ 10 g/m^2^)	No (β 0.38; -1.13–1.90), >10 (β -0.85; -2.91–1.22)	
			Ifosfamide (≤ 10 g/m^2^)	No (β 0.54; −2.89–3.96), > 10 (β 0.66; −3.06–4.39)	
			RT exposure (none)	**Thorax (β** − **3.67;** − **5.54 −** − **1.79),** Abdomen (β − 3.54; -5.87− − 1.20), Spine (β − − 0.79; − 2.92–1.24), total body (β − 0.53; − 4.01–2.94)	
		FS < 30% (27%)	Male sex	OR 0.73; 0.47–1.13	
			Age at diagnosis (> 15 years)	**0–5 (OR 2.94; 1.08–8.02), > 5–10 (OR 1.64; 0.67– 4.01), > 10–15 (OR 1.45; 0.64–3.28)**^**b**^	
			Time since diagnosis (5–10 years)	**10–15 (OR 0.80; 0.41–1.54), 15–20 (OR 0.40; 0.18–0.86), 20–25 (OR 0.48; 0.19–1.23), > 25 (OR 0.11; 0.03–0.42)**^**b**^	
			Vincristine exposure	OR 1.47; 0.71–3.05	
			Anthracycline dose (0–150 mg/m^2^)	**151–300 (OR 3.98; 1.58–10.01), 301–450 (OR 7.77; 2.85–21.22), > 450 (OR 10.58; 3.35–33.40)**^**b**^	
			Cyclophosphamide (≤ 10 g/m^2^)	No (OR 1.01; 0.52–1.99), > 10 (OR 1.01; 0.45–2.26)	
			Ifosfamide (≤ 10 g/m^2^)	No (OR 1.25; 0.23–6.67), > 10 (OR 1.50; 0.26–8.82)	
			RT exposure (none)	**Thorax (OR 3.49; 1.60–7.61),** Abdomen (OR 2.66; 1.00–7.05), Spine (OR 0.64; 0.23–1.74), total body (OR 0.53; 0.10–2.87)	
Hudson, 2007 [21]	*n* = 278 various cancers, no congenital heart disease/congestive heart failure/chronic illness/trisomy21/anaemia	FS (continuous)	Age at diagnosis	< 5 years (mean 35%), ≥ 5 years (mean 32%)	22% had no anthracycline exposure
			Diagnosis group	Leukaemia (mean 36%), Sarcoma (mean 32%), Lymphoma (mean 33%), Embryonal (mean 34%)	
			QTc time	Normal (mean 34%), prolonged (mean 29%)	
			Years off therapy per 5-year increment	β **−**.004	
			Anthracycline dose per 50 mg/m^2^ increment	**β −.008**	
		FS < 28% (14%)	Age at diagnosis (< 5 years)	≥ 5 (OR 2.41; 0.91–6.40)	
			Diagnosis group (leukaemia)	**Sarcoma (OR 5.09; 1.30–19.89),** Lymphoma (OR 2.04; 0.47–8.94), Embryonal (OR 1.70; 0.36–8.04)	
			Years off therapy per 5-year increment	OR 1.08; 0.52–2.27	
			Anthracycline dose per 50 mg/m^2^ increment	**OR 1.19; 1.01–1.39**	
Pein, 2004 [22]	*n* = 205 any cancer, anthracycline, diagnosis + ≥ 15 years	FS (continuous)	Anthracycline dose	**≤ 150 mg/m**^**2**^ **(mean 35%), 151–250 (mean 34%), 251–400 (mean 33%), > 400 (mean 30%)**^**b**^	
		Teich LVEF (continuous)	Anthracycline dose	**≤ 150 mg/m**^**2**^ **(mean 64%), 151–250 (mean 62%), 251–400 (mean 61%), > 400 (mean 57%)**^**b**^	

Bolded values indicate statistical significance in multivariable analysis that at least included sex, age at diagnosis and either attained age or follow-up time since cancer diagnosis. ^a^Included, but no effect size reported for multivariable model; ^b^significant trend

*FS*, fractional shortening; *GCS*, global circumferential strain; *GLS*, global longitudinal strain; *LVEF*, left ventricular ejection fraction; *OR*, odds ratio; *Heart RT*, radiotherapy involving the heart region; *RR*, risk ratio; n.m., not mentioned

## Discussion

This systematic review shows a high variation in the prevalence of ALVSD in long-term CCS, also when including contemporary echocardiographic measurements such as myocardial strain. The heterogeneity in cardiotoxic exposure and time since diagnosis, within and between cohorts, as well as heterogeneous measurement methods and cut-off values for abnormality, prevented pooling of data. This makes large cohort studies and pooling of individual patient data the most appropriate ways to study the epidemiology of ALVSD in long-term CCS. The prevalence of abnormal GLS is higher compared to abnormal LVEF, and both are increased in studies with longer periods of follow-up. The reviewed studies add data to the conclusions from our previous review on the increased risk of ALVSD with higher doses of cardiotoxic exposures [5]. However, for additional risk factors that could aid further risk stratification, the studies show little agreement.

### Prevalence of ALVSD

Within two studied cohorts, GLS-based ALVSD was more prevalent than LVEF-based ALVSD (9% versus 1%, and 30% versus 6%, respectively), at a median of one to two decades after diagnosis [13, 18]. Although the CCS studied by Christiansen et al. did not all receive anthracyclines, they found prevalences of abnormal GLS (32%) and either abnormal LVEF or FS (11%), at a mean of 22 years since diagnosis, that were in accordance with the included studies [17]. Strikingly, for CCS at median ages of 20 to 31 years, these four to five times greater prevalences of GLS-based ALVSD versus LVEF-based ALVSD, approximate those in a> 80 years old subgroup of a United States community-based cohort[26].

Ageing is an important risk factor for cardiovascular disease in the general population. The highest prevalence of ALVSD indeed was reported in cohorts with the longest follow-up since diagnosis, but not all included risk factor analyses support this finding.

### Risk factors for ALVSD

Cumulative anthracycline dose and radiotherapy involving the heart region are evident risk factors for ALVSD, across echocardiographic parameters. Even the lowest anthracycline dose categories carry a risk of ALVSD [18]. Interestingly, in the largest included study, the risk ratios for abnormal GLS were only slightly elevated in the higher dose categories (up to 1.73), compared to the straightforward increasing risk for abnormal LVEF up to 7.71 [18]. This may reflect a higher prevalence of abnormal GLS among CCS with no anthracycline exposure. These CCS were, in this study, exposed to radiotherapy involving the heart region. Reporting systolic function parameters as continuous outcomes might allow to find the lowest cardiotoxic doses and takes the degree of abnormality into account in risk factor analyses.

There was no agreement on the role of sex, age at cancer diagnosis or attained age as risk factors for ALVSD. Interestingly, Armstrong et al. found more abnormal LVEF in males but more abnormal GLS in females [18]. Since males are known to have lower LVEF values [9], this perceived discrepancy might dissolve after application of sex-specific LVEF cut-off values, as was already done for GLS. Studies on clinical heart failure incidence also remain ambiguous on the role of female sex as a risk factor [2, 27].

The largest included study investigated the association of ALVSD with modifiable cardiovascular risk factors. The authors found all components of the metabolic syndrome associated with abnormal GLS and hypertension associated with abnormal LVEF [18]. This substantiates the evidence provided by large cohort studies that assess risk factors for clinical heart failure in CCS [28, 29], indicating especially hypertension as clinically actionable risk factor.

### Comparison of different echocardiographic parameters

Abnormal GLS is regarded as an early and sensitive indicator of systolic dysfunction in adults with cardiovascular disease, including adult cardio-oncology patients [6, 7, 30]. As expected, abnormal GLS was more prevalent than abnormal LVEF within our included cohorts. However, GLS measurement should not replace LVEF, since not only longitudinal shortening contributes to LVEF but also circumferential shortening, wall thickness and end-diastolic volume [31]. This may also explain why some subjects with abnormal LVEF exhibited normal GLS [17, 19]. Combined measurements may add prognostic value to single measurements.

A systematic review found that GCS abnormalities were more consistently present than GLS abnormalities in CCS at longer follow-up after anthracycline therapy. It also showed, with some heterogeneity, that GLS abnormalities were more frequent in the first year posttreatment [32]. In our review, only one of two studies showed a higher prevalence of abnormal GCS than of abnormal GLS [19]. Since the reproducibility of GCS measurements is questionable, GCS may be less useful as a sensitive marker for ALVSD [18, 24].

Different contraction and remodelling patterns, which might be caused by different cardiotoxic exposures, affect different parameters of systolic function. Furthermore, prevalence of abnormality is affected by the definition of abnormality, including measurement method and cut-off value. In the present review, the prevalence of abnormal FS, when defined with a liberal cut-off value of < 30%, approximates that of an abnormal GLS, albeit in different cohorts [18, 20, 21]. However, GLS was shown to better correlate with LVEF than with FS [19]. Ideally, the relationships of systolic function parameters and cut-off values should be studied within large cohorts that include a control group, to put the abnormality in perspective.

### Which systolic function parameter to use?

Different LV function parameters may serve different purposes, such as selecting CCS that would benefit from therapy, or identification of CCS with very low risk of future heart failure. Prognostic evidence for echocardiographic parameters was only recently presented with retrospective data on longitudinal changes of LVEF and FS [33], and the 10-year predictive value of LVEF measurement, when added to anthracycline dose and radiotherapy, for developing an LVEF < 40% [34].

Regarding GLS, the recently published results on GLS-guided cardioprotection in adults on active cancer treatment do not justify early initiation of heart failure treatment [35]. However, the evidence on the added sensitivity and prognostic value of GLS over LVEF in predicting severe endpoints is accumulating in cardiology and adult cardio-oncology [6, 7]. The lack of evidence in CCS should not be confused with lack of prognostic value. Knowing this, research may focus on strict cardiovascular risk management in CCS with abnormal GLS, and surveillance reduction for those with normal GLS.

The current cardiomyopathy surveillance guideline describes LVEF, FS and wall stress as ‘most frequently used and readily reproducible variables of LV systolic function’[4]. It should be noted that linear measurements of global LV function, such as FS, are discouraged in adult guidelines for echocardiography [9]. Linear measurements may also be inferior to volumetric methods in children [36]. They ignore regional wall motion abnormalities and abnormal ventricular geometry, which may not be uncommon in CCS since cardiotoxicity can include valvular and ischaemic heart disease [3].

Also, 3D LVEF measurement is more reproducible than biplane LVEF [37], which is useful in detecting subtle changes during follow-up. It is also more comparable to magnetic resonance imaging as gold standard [38]. Multi-view GLS measurements are considered more reproducible than measurements in a single apical view [39].

Echocardiography labs incorporating GLS measurement in their clinical routine will facilitate future studies. GLS measurement has been standardized by recommendations of a dedicated task force [40]. Practical cut-off values were proposed in adult cardio-oncology patients with an LVEF of 50–59%, with − 16% as most specific cut-off for abnormal without losing sensitivity. Values between − 16 and − 18% constitute a ‘grey zone’, which can be acceptable in elderly subjects with hypertension but abnormal in healthy young adults [6, 41]. These cut-off values are not yet validated in pediatric subjects.

### Strengths and limitations

Studies carried an unknown risk of selection bias and a substantial risk of detection bias and reporting bias, the latter hampering detailed comparison of heterogeneous cohorts. Large within-study variation in important study characteristics always prevents pooling of results. We chose rather stringent inclusion criteria, as small studies would be underpowered to estimate prevalences [8]. Prevalence estimation was not the primary goal of many potentially eligible studies. No multivariable risk factor analysis exactly matched our inclusion criteria, but all adequately adjusted for the most important confounders. Our attempts to contact study authors made new data available, to construct a complete as possible review. Narrowing down the inclusion criteria to specific cut-off values for ALVSD would result in missing information. We highlight that the prevalence of ALVSD is related to the definition used, underscoring the need to harmonize ALVSD definitions in CCS.

## Conclusions

ALVSD detected with echocardiography is common in long-term CCS treated with anthracyclines. GLS identifies a higher prevalence of ALVSD, compared to LVEF, but should not replace LVEF measurement. Even CCS treated with the lowest anthracycline doses may show ALVSD. Hypertension might be an important modifiable risk factor for ALVSD. The diagnostic and prognostic value of GLS, as well as the relations between different echocardiographic measurements, should be evaluated within large cohorts.

## Supplementary information

ESM 1 (DOCX 299 kb)

## Data Availability

All data relevant to the study are included in the article or uploaded as supplementary information.
