# Supplementary material for: Asymptomatic systolic dysfunction on contemporary echocardiography in anthracycline-treated long-term childhood cancer survivors: a systematic review
Source: J Cancer Surviv. 2021 Mar 27;16(2):338–52. doi: 10.1007/s11764-021-01028-4 (PMC8964593; doi:10.1007/s11764-021-01028-4)
Supplement: Supplementary file 1 — (DOCX 299 kb) [file 11764_2021_1028_MOESM1_ESM.docx]

| **Table S1.** Full search strategy | | |
| --- | --- | --- |
| **PubMed** | | |
| **1. Anthracycline therapy** | "Anthracyclines"[Mesh] OR "Antineoplastic Agents/adverse effects"[Mesh] OR "Neoplasms/drug therapy"[Mesh] OR "Cardiotoxins"[Mesh] OR anthracyclin*[tiab] OR antracyclin*[tiab] OR doxorubic*[tiab] OR dox sl[tiab] OR daunorubic*[tiab] OR NSC 82151[tiab] OR demethoxydaunorubic*[tiab] OR IMI 30[tiab] OR IMI30[tiab] OR NSC 256439[tiab] OR idarubic*[tiab] OR epidoxorubic*[tiab] OR epi dxr[tiab] OR NSC 256942[tiab] OR IMI 28[tiab] OR IMI28[tiab] OR epirubic*[tiab] OR adriamyc*[tiab] OR epiadriamyc*[tiab] OR plicamyc*[tiab] OR farmorubic*[tiab] OR rubidomyc*[tiab] OR rubomyc*[tiab] OR daunomyc*[tiab] OR adriablastin*[tiab] OR adriblastin*[tiab] OR cerubidin*[tiab] OR daunoblastin*[tiab] OR daunoxom*[tiab] OR daunosom*[tiab] OR doxil[tiab] OR caelyx[tiab] OR myocet[tiab] OR cardiotoxin*[tiab] | |
| **2.**  **Children** | "Child"[Mesh] OR "Infant"[Mesh] OR "Adolescent"[Mesh] OR "Pediatrics"[Mesh] OR "Minors"[Mesh] OR "Young Adult"[Mesh] OR child*[tiab] OR infant*[tiab] OR baby[tiab] OR babies[tiab] OR newborn*[tiab] OR new-born*[tiab] OR neonat*[tiab] OR perinat*[tiab] OR toddler*[tiab] OR adolescen*[tiab] OR pubescen*[tiab] OR teen*[tiab] OR youth*[tiab] OR young age[tiab] OR minors*[tiab] OR pediatric*[tiab] OR paediatric*[tiab] OR juvenil*[tiab] OR boy[tiab] OR boyhood[tiab] OR girl*[tiab] OR schoolchild*[tiab] OR school child*[tiab] OR kid[tiab] OR kids[tiab] OR young male[tiab] OR young female[tiab] OR young adult*[tiab] OR young men[tiab] OR young woman[tiab] | |
| **3.**  **Asymptomatic systolic dysfunction** | "Heart/adverse effects"[Mesh] OR "Heart/toxicity"[Mesh] OR "Ventricular Dysfunction"[Mesh] OR "Cardiotoxicity"[Mesh] OR cardiotoxicit*[tiab] OR ejection fraction[tiab] OR LVEF[tiab] OR "Stroke Volume"[Mesh] OR contractilit*[tiab] OR "Cardiomyopathies"[Mesh] OR cardiomyopath*[tiab] OR ((cardiac[tiab] OR myocard*[tiab] OR heart[tiab] OR ventricular[tiab] OR systolic[tiab]) AND (damage[tiab] OR injur*[tiab] OR toxicit*[tiab] OR disease*[tiab] OR dysfunct*[tiab] OR function[tiab] OR strain[tiab]) OR deform*[tiab] OR speckle track*[tiab] OR 2DSTE[tiab] OR "Echocardiography"[Mesh] OR echocardiograph*[tiab] OR fractional shortening[tiab] OR shortening fraction[tiab] | |
|  | 1 AND 2 AND 3 NOT ("Animals"[Mesh] NOT "Humans"[Mesh]) | |
| **EMBASE** | | |
| **1. Anthracycline therapy** | 1. exp antineoplastic antibiotic/  2. exp cancer chemotherapy/  3. exp cardiotoxin/ or cardiotoxin$.ti,ab,kw. 4. (anthracyclin$ or antracyclin$).ti,ab,kw. 5. (daunoxom$ or daunosom$ or doxil or caelyx or myocet or doxorubic$).ti,ab,kw. 6. (NSC-82151 or NSC 82151 or NSC82151).ti,ab,kw. 7. (cerubidin$ or daunoblastin$ or daunorubic$).ti,ab,kw. 8. (dauno-rubidomyc$ or rubidomyc$ or rubomyc$ or daunomyc$).ti,ab,kw.  9. (adramyc$ or adriamyc$ or adriablastin$ or adriblastin$ or DOX-SL or DOX SL).ti,ab,kw. | 10. (IMI-28 or IMI 28 or IMI28 or NSC256942 or NSC-256942 or NSC 256942).ti,ab,kw. 11. (epirubic$ or farmorubic$).ti,ab,kw. 12. (4-epi-adriamy$ or epiadriamyc$ or 4-epiadriamy$ or 4-epi-DXR or DXR).ti,ab,kw. 13. (NSC 256439 or NSC-256439 or NSC256349 or IMI30 or IMI 30 or IMI-30 or idarubic$).ti,ab,kw. 14. (4-demethoxydaunorubi$ or demethoxydaunorubic$ or 4-desmethoxydaunorubi$ or desmeth oxydaunorubic$).ti,ab,kw. 15. or/1-14 |
| **2.**  **Children** | 1. infan$.ti,ab,kw. 2. (newborn$ or new-born$).ti,ab,kw. 3. (perinat$ or neonat$).ti,ab,kw. 4. exp childhood/ 5. (baby$ or babies).ti,ab,kw. 6. toddler$.ti,ab,kw. 7. minors$.ti,ab,kw. 8. (boy or boys or boyhood).ti,ab,kw. 9. girl$.ti,ab,kw. 10. (kid or kids).ti,ab,kw. 11. exp child/ 12. child$.ti,ab,kw. | 13. school child/ or school$.ti,ab,kw. 14. (schoolchild$ or school child$).ti,ab,kw. 15. (adolescen$ or youth$ or young age or teen$).ti,ab,kw. 16. (juvenil$ or under$age$).ti,ab,kw. 17. pubescen$.ti,ab,kw. 18. exp pediatrics/ or p?ediatric$.ti,ab,kw. 19. (prematur$ or preterm$).ti,ab,kw. 20. exp young adult/ or young adult$.ti,ab,kw. 21. (young wom#n or young men or young male or young female).ti,ab,kw. 22. exp childhood cancer survivor/  23. or/1-22 |
| **3.**  **Asymptomatic systolic dysfunction** | 1. exp heart injury/  2. exp cardiotoxicity/ or cardiotoxicit$.ti,ab,kw. 3. exp cardiomyopathy/ or cardiomyopath$.ti,ab,kw.  4. ((myocard$ or heart or ventricular or cardiac or systolic) and (damage or injur$ or disease$ or dysfunct$ or function or toxicit$ or strain)).ti,ab,kw. 5. exp heart function/ or (ejection fraction or LVEF or contractil$).ti,ab,kw. | 6. exp echocardiography/ or echocardiograph$.ti,ab,kw. 7. (speckle track$ or 2DSTE or deform$).ti,ab,kw. 8. (fractional shortening or shortening fraction).ti,ab,kw. 9. or/1-8 |
|  | 1 and 2 and 3 NOT ((exp animal/ or nonhuman/) NOT exp human/) NOT (‘editorial’ or ‘conference review’ or ‘conference paper’ or ‘conference abstract’)[Publication type] | |
| **Cochrane CENTRAL** | | |
| **1. Anthracycline therapy** | MeSH descriptor: [Anthracyclines] explode all trees OR MeSH descriptor: [Cardiotoxins] explode all trees OR MeSH descriptor: [Antineoplastic Agents] explode all trees and with qualifier(s): [adverse effects - AE] OR MeSH descriptor: [Neoplasms] explode all trees and with qualifier(s): [drug therapy - DT] OR (anthracyclin* OR antracyclin* OR doxorubic* OR dox OR daunorubic* OR NSC82151 OR 82151 OR demethoxydaunorubic* OR IMI30 OR NSC256439 OR 256439 OR idarubic* OR epidoxorubic* OR dxr OR NSC256942 OR 256942 OR IMI28 OR epirubic* OR adriamyc* OR epiadriamyc* OR plicamyc* OR farmorubic* OR rubidomyc* OR rubomyc* OR daunomyc* OR adriablastin* OR adriblastin* OR cerubidin* OR daunoblastin* OR daunoxom* OR daunosom* OR doxil OR caelyx OR myocet OR cardiotoxin*):ti,ab,kw | |
| **2.**  **Children** | MeSH descriptor: [Child] explode all trees OR MeSH descriptor: [Infant] explode all trees OR MeSH descriptor: [Adolescent] explode all trees OR MeSH descriptor: [Pediatrics] explode all trees OR MeSH descriptor: [Minors] explode all trees OR MeSH descriptor: [Young Adult] explode all trees OR "Young Adult"[Mesh] OR (child* OR infant* OR baby OR babies OR newborn* OR new-born* OR neonat* OR perinat* OR toddler* OR adolescen* OR pubescen* OR teen* OR youth* OR young age OR minors* OR pediatric* OR paediatric* OR juvenil* OR boy OR boyhood OR girl* OR schoolchild* OR school child* OR kid OR kids OR young male OR young female OR young adult* OR young men OR young woman):ti,ab,kw | |
| **3.**  **Asymptomatic systolic dysfunction** | MeSH descriptor: [Ventricular Dysfunction] explode all trees OR MeSH descriptor: [Cardiotoxicity] explode all trees OR MeSH descriptor: [Cardiomyopathies] explode all trees OR MeSH descriptor: [Stroke Volume] explode all trees OR MeSH descriptor: [Echocardiography] explode all trees OR (cardiotoxicit* OR ejection fraction OR LVEF OR contractilit* OR cardiomyopath* OR deform* OR speckle track* OR 2DSTE OR echocardiograph* OR fractional shortening OR shortening fraction OR ((ventricular OR cardiac OR systolic OR heart OR myocard*) AND (damage OR injury OR disease* OR function OR dysfunct* OR strain OR toxicit*))):ti,ab,kw | |
|  | *Limit: trials* 1 AND 2 AND 3 NOT (MeSH descriptor: [Animals] explode all trees NOT MeSH descriptor: [Humans] explode all trees) | |

| **Table S2.** Risk of bias assessment criteria | | |
| --- | --- | --- |
|  | **Internal validity** | **External validity** |
| **Study group** | **Selection bias (representative: yes/no)**  • if the described study group consisted of more than 95% of the original cohort of asymptomatic 5-year survivors of childhood cancer treated with anthracyclines with(out) radiotherapy on the heart region   • or if it was a random sample with respect to the cancer treatment | **Reporting bias (well‐defined: yes/no)**  • if the mean, the median or the range of the cumulative anthracycline, other potential cardiotoxic treatment (radiotherapy, mitoxantrone, ifosfamide, cyclophosphamide and vincristine) was mentioned and prior cancer treatment (++).   • if only the mean, the median or the range of the cumulative anthracycline and irradiation dose was mentioned (+) |
| **Follow-up** | **Attrition bias (adequate: yes/no)**  • if the outcome was assessed for more than 95% of the study group of interest (++)   • or if the outcome was assessed for 65‐95% of the study group of interest (+) | **Reporting bias (well‐defined: yes/no)**  • if the length of follow‐up was mentioned |
| **Outcome** | **Detection bias (blind: yes/no)**  • if the outcome assessors were blinded to the investigated determinant | **Reporting bias (well‐defined: yes/no)**  • if clearly defined cut-off values were used according to guidelines, controls or vendor-specific normative values |
| **Risk estimation** | **Confounding (adjustment for other factors: yes/no)**  • if important prognostic factors (i.e. sex, age at diagnosis, co‐treatment) and either attained age or follow‐up from diagnosis were taken adequately into account | **Analyses (well‐defined: yes/no)**  • if a risk ratio, odds ratio, attributable risk, linear or logistic regression model, mean difference or Chi² was calculated |

| **Table S3.** Reasons for exclusion of 152 studies (multiple reasons per study possible) | |
| --- | --- |
| **Reason for exclusion (n)** | **References** |
| Not original study (28) | (1-28) |
| Conference abstract (4) | (29-32) |
| Case report (3) | (33-35) |
| Wrong study design / selected population (6) | (36-41) |
| Adults / not specific on children (16) | (42-57) |
| Asymptomatic status not mentioned (5) | (58-62) |
| Not specific on anthracyclines (7) | (61, 63-68) |
| Follow-up too short / unclear (49) | (49, 51, 54, 58, 59, 61, 65, 66, 69-109) |
| Too small cohort (22) | (75, 81-83, 86, 91, 93, 101, 108, 110-122) |
| Wrong outcome / not defined (41) | (39-41, 43, 44, 46, 57, 58, 60, 62, 66, 68, 69, 77, 81, 92, 94, 96, 98, 102-104, 108, 109, 117, 123-138) |
| Unable to obtain full-text / translation (4) | (139-141) / (142) |
| Overlapping cohorts (14) | (67, 70, 106, 107, 136, 143-151) |
| Authors contacted: no response, refusal or invalid (25) | (57, 60-62, 65, 66, 68, 70, 96-100, 102-105, 109, 134-136, 142, 144, 152) |

**References to excluded studies**

1. Aminkeng F, Ross CJD, Rassekh SR, Rieder MJ, Bhavsar AP, Sanatani S, et al. Pharmacogenomic screening for anthracycline-induced cardiotoxicity in childhood cancer. British Journal of Clinical Pharmacology. 2017;83(5):1143-5.

2. Armenian S, Lenihan D. Childhood Cancer Survivorship, Late Cardiotoxicity, and CV Prevention. OnCOReview. 2016;6(3):A100-2.

3. Armenian SH, Wong FL. Screening for Anthracycline-Related Cardiac Dysfunction in Childhood Cancer Survivors: Can Less be More? Pediatric Blood and Cancer. 2015;62(12):2067-8.

4. Bhatia S. Long-term complications of therapeutic exposures in childhood: Lessons learned from childhood cancer survivors. Pediatrics. 2012;130(6):1141-3.

5. Doz F. Late effects of cancer chemotherapy in children. [French]. Archives de Pediatrie. 2007;14(6):612-4.

6. Ehrhardt MJ, Armenian SH, Fulbright JM. Screening and intervention for treatment-related cardiac dysfunction in childhood cancer survivors. Future Oncology. 2015;11(14):2031-4.

7. Ghosh J, Bajpai J. Chemotherapy for osteosarcoma: Adverse effects and remedial measures. Pediatric Hematology Oncology Journal. 2017;2(2):41-7.

8. Goey AKL, Schellens JHM, Beijnen JH, Huitema ADR. Dexrazoxane in anthracycline-induced cardiotoxicity and anthracycline extravasation. [Dutch]. Nederlands Tijdschrift voor Geneeskunde. 2010;154(23):1105-10.

9. Gores G, Ravekes W, Koestenberger M. Relevance of tricuspid annular peak systolic velocity (S') to detect systolic right-ventricular impairment after anthracycline cancer treatment in childhood. Pediatric Cardiology. 2014;35(1):188.

10. Green DM. Late effects of treatment for cancer during childhood and adolescence. Current Problems in Cancer. 2003;27(3):127-42.

11. Hudson MM. Anthracycline cardiotoxicity in long-term survivors of childhood cancer: The light is not at the end of the tunnel. Pediatric Blood and Cancer. 2007;48(7):649-50.

12. Lipshultz SE, Alvarez JA, Scully RE. Anthracycline associated cardiotoxicity in survivors of childhood cancer. Heart. 2008;94(4):525-33.

13. Lipshultz SE, Franco VI, Cochran TR. Cardiotoxicity in childhood cancer survivors: A problem with long-term consequences in need of early detection and prevention. Pediatric Blood and Cancer. 2013;60(9):1395-6.

14. Moon MA. Considerable' subclinical CV disease in childhood cancer survivors. Oncology Report. 2016;12(2):34.

15. Robison LL, Bhatia S. Late-effects among survivors of leukaemia and lymphoma during childhood and adolescence. British Journal of Haematology. 2003;122(3):345-59.

16. Schwartz CL, Constine LS, London WB, Sposto R, Friedman D, Tebbi CK, et al. In reply [2]. Journal of Clinical Oncology. 2007;25(21):3180.

17. Silber JH. Can dexrazoxane reduce myocardial injury in anthracycline-treated children with acute lymphoblastic leukemia? Nature Clinical Practice Oncology. 2004;1(1):16-7.

18. Bergler-Klein J. Right from the heart: survivors of childhood cancer and the right ventricle. European heart journal cardiovascular Imaging. 2016;17(7):742-3.

19. Burch M. Heart failure in the young. Heart. 2002;88(2):198-202.

20. Candela JL. CE: Cardiotoxicity and Breast Cancer as Late Effects of Pediatric and Adolescent Hodgkin Lymphoma Treatment. The American journal of nursing. 2016;116(4):32-42; quiz 3.

21. Ewer MS. Exercise echocardiography reflects cumulative anthracycline exposure during childhood. Pediatric blood & cancer. 2004;42(7):554-5.

22. Hashimoto I, Ichida F. Left Ventricular Fiber Structure and Myocardial Deformation for Assessment of Cardiotoxicity. Circulation journal : official journal of the Japanese Circulation Society. 2018;82(3):648-9.

23. Lange BJ, Woods WG. Ya gotta have heart. Pediatric blood & cancer. 2011;56(4):513-4.

24. Rosenberg H. Cardiac function in 5-year survivors of childhood cancer. Archives of internal medicine. 2011;171(3):264; author reply -5.

25. van Dalen EC, van der Pal HJ, van den Bos C, Caron HN, Kremer LC. Treatment for asymptomatic anthracycline-induced cardiac dysfunction in childhood cancer survivors: the need for evidence. Journal of clinical oncology : official journal of the American Society of Clinical Oncology. 2003;21(17):3377; author reply -8.

26. York A. No more breaking hearts. Lancet Oncology. 2004;5(8):460.

27. Acar Z, Kale A, Turgut M, Demircan S, Durna K, Demir S, et al. Efficiency of atorvastatin in the protection of anthracycline-induced cardiomyopathy. J Am Coll Cardiol. 2011;58(9):988-9.

28. Steingart RM, Liu JE, Oeffinger KC. Cost-effectiveness of screening for asymptomatic left ventricular dysfunction in childhood cancer survivors. Ann Intern Med. 2014;160(10):731-2.

29. Aggarwal S, Chow EJ, Sasaki N, Doody DR, Armenian SH, Asselin BL, et al. Long-term cardioprotective effects of dexrazoxane infusion during anthracycline chemotherapy: a children's oncology group speckle echocardiography study. Journal of the american society of echocardiography. 2018;31(6):B14‐B5.

30. Hyoungsoo C, Hyoung Jin K, Hee Young S, Jong Jin S, Hyo Seop A, Jung Yun C. Dexrazoxane for protection of anthracycline cardiotoxicity in children with solid tumors. Pediatric blood & cancer. 2009;53(5 (41th Annual Conference of the International Society of Paediatric Oncology, SIOP, Sao Paulo, Brazil)):735‐6 [Abstract O.083].

31. Taha FA. Implication of two-dimensional speckle tracking deformation imaging in early detection and management of children underwent anthracycline induced cardiotoxicity. European heart journal, supplement Conference: 2017 cardioalex conference Egypt. 2017;19(Supplement G):G6‐G7.

32. Getz KD, Sung L, Gerbing RB, Alonzo TA, Li Y, Huang YS, et al. Occurrence and resolution of anthracycline cardiotoxicity and impact on treatment outcomes among children treated on the AAML1031 clinical trial: a report from the children's oncology group. Blood. 2019;134.

33. Guendouz S, Buicuic O, Kirsch M, Benaiem N, Poulard JE, Deux JF, et al. Restrictive cardiomyopathy associated with left ventricle and left atria endocardial calcifications following chemotherapy. Journal of the American College of Cardiology. 2011;57(15):1633.

34. Singh HR, Paules M, Forbes TJ, Zilberman MV. Reversible dilated cardiomyopathy and neuroblastoma. The Journal of pediatrics. 2005;147(2):271.

35. Trebo MM, Mann G, Dworzak M, Zoubek A, Gadner H. Wilms Tumor and Cardiomyopathy. Medical and Pediatric Oncology. 2003;41(6):574.

36. Mansouri I, Allodji RS, Hill C, El-Fayech C, Pein F, Diallo S, et al. The role of irradiated heart and left ventricular volumes in heart failure occurrence after childhood cancer. European Journal of Heart Failure. 2018.

37. Cifra B, Chen CK, Fan CPS, Slorach C, Manlhiot C, McCrindle BW, et al. Dynamic Myocardial Response to Exercise in Childhood Cancer Survivors Treated with Anthracyclines. Journal of the American Society of Echocardiography. 2018;31(8):933-42.

38. Hildebrandt MAT, Reyes M, Wu X, Pu X, Thompson KA, Ma J, et al. Hypertension Susceptibility Loci are Associated with Anthracycline-related Cardiotoxicity in Long-term Childhood Cancer Survivors. Scientific reports. 2017;7(1):9698.

39. Visscher H, Rassekh SR, Sandor GS, Caron HN, Van Dalen EC, Kremer LC, et al. Genetic variants in SLC22A17 and SLC22A7 are associated with anthracycline-induced cardiotoxicity in children. Pharmacogenomics. 2015;16(10):1065-76.

40. Visscher H, Ross CJD, Rassekh SR, Barhdadi A, Dube MP, Al-Saloos H, et al. Pharmacogenomic prediction of anthracycline-induced cardiotoxicity in children. Journal of Clinical Oncology. 2012;30(13):1422-8.

41. Visscher H, Ross CJD, Rassekh SR, Sandor GSS, Caron HN, van Dalen EC, et al. Validation of variants in SLC28A3 and UGT1A6 as genetic markers predictive of anthracycline-induced cardiotoxicity in children. Pediatric Blood and Cancer. 2013;60(8):1375-81.

42. Hamirani Y, Fanous I, Kramer CM, Wong A, Salerno M, Dillon P. Anthracycline- and trastuzumab-induced cardiotoxicity: a retrospective study. Medical Oncology. 2016;33 (7) (no pagination)(82).

43. Jurczak W, Szmit S, Sobocinski M, Machaczka M, Drozd-Sokolowska J, Joks M, et al. Premature cardiovascular mortality in lymphoma patients treated with (R)-CHOP regimen - A national multicenter study. International Journal of Cardiology. 2013;168(6):5212-7.

44. Krul IM, Opstal-van Winden AWJ, Janus CPM, Daniels LA, Appelman Y, Maas AHEM, et al. Cardiovascular Disease Risk After Treatment-Induced Premature Ovarian Insufficiency in Female Survivors of Hodgkin Lymphoma. Journal of the American College of Cardiology. 2018;72(25):3374-5.

45. Limat S, Daguindau E, Cahn JY, Nerich V, Brion A, Perrin S, et al. Incidence and risk-factors of CHOP/R-CHOP-related cardiotoxicity in patients with aggressive non-Hodgkin's lymphoma. Journal of Clinical Pharmacy and Therapeutics. 2014;39(2):168-74.

46. Longhi A, Ferrari S, Tamburini A, Luksch R, Fagioli F, Bacci G, et al. Late effects of chemotherapy and radiotherapy in osteosarcoma and Ewing sarcoma patients: The Italian Sarcoma Group Experience (1983-2006). Cancer. 2012;118(20):5050-9.

47. Aviles A, Neri N, Nambo JM, Huerta-Guzman J, Talavera A, Cleto S. Late cardiac toxicity secondary to treatment in Hodgkin's disease. A study comparing doxorubicin, epirubicin and mitoxantrone in combined therapy. Leukemia & lymphoma. 2005;46(7):1023-8.

48. Busia A, Laffranchi A, Viviani S, Bonfante V, Villani F. Cardiopulmonary toxicity of different chemoradiotherapy combined regimens for Hodgkin's disease. Anticancer research. 2010;30(10):4381-7.

49. Chaladze T, Megreladze I, Zodelava M. Early cardiotoxicity induced by treatment of hematologic malignancies and the risk-factors of its manifestation. Georgian medical news. 2005(129):117-9.

50. Fridrik MA, Jaeger U, Petzer A, Willenbacher W, Keil F, Lang A, et al. Cardiotoxicity with rituximab, cyclophosphamide, non-pegylated liposomal doxorubicin, vincristine and prednisolone compared to rituximab, cyclophosphamide, doxorubicin, vincristine, and prednisolone in frontline treatment of patients with diffuse large B-cell lymphoma: A randomised phase-III study from the Austrian Cancer Drug Therapy Working Group [Arbeitsgemeinschaft Medikamentose Tumortherapie AGMT](NHL-14). European journal of cancer (Oxford, England : 1990). 2016;58:112-21.

51. Liu Y, Ke XY, Ma J, Shen ZX, Zhang XH, Du X, et al. [Multicenter randomized control trial on safety of domestic idarubicin for acute leukemia]. Zhonghua zhong liu za zhi [Chinese journal of oncology]. 2006;28(9):706-8.

52. Saito Y, Susukida I, Uzuka Y, Kanai H. Noninvasive early detection of anthracycline-induced cardiotoxicity in patients with hematologic malignancies using the phased tracking method. Cancer medicine. 2016;5(9):2276-85.

53. Wethal T, Lund MB, Edvardsen T, Foss SD, Pripp AH, Holte H, et al. Valvular dysfunction and left ventricular changes in Hodgkin's lymphoma survivors. A longitudinal study. British Journal of Cancer. 2009;101(4):575-81.

54. Yu AF, Raikhelkar J, Zabor EC, Tonorezos ES, Moskowitz CS, Adsuar R, et al. Two-Dimensional Speckle Tracking Echocardiography Detects Subclinical Left Ventricular Systolic Dysfunction among Adult Survivors of Childhood, Adolescent, and Young Adult Cancer. BioMed Research International. 2016;2016 (no pagination)(9363951).

55. Cardinale D, Sandri MT, Colombo A, Colombo N, Boeri M, Lamantia G, et al. Prognostic value of troponin I in cardiac risk stratification of cancer patients undergoing high-dose chemotherapy. Circulation. 2004;109(22):2749-54.

56. Cardinale D, Colombo A, Bacchiani G, Tedeschi I, Meroni CA, Veglia F, et al. Early detection of anthracycline cardiotoxicity and improvement with heart failure therapy. Circulation. 2015;131(22):1981-8.

57. Getz KD, Sung L, Ky B, Gerbing RB, Leger KJ, Leahy AB, et al. Occurrence of Treatment-Related Cardiotoxicity and Its Impact on Outcomes Among Children Treated in the AAML0531 Clinical Trial: A Report From the Children's Oncology Group. Journal of clinical oncology : official journal of the American Society of Clinical Oncology. 2019;37(1):12-21.

58. Hines MR, Mulrooney DA, Hudson MM, Ness KK, Green DM, Howard SC, et al. Pregnancy-associated cardiomyopathy in survivors of childhood cancer. Journal of cancer survivorship : research and practice. 2016;10(1):113-21.

59. Aminkeng F, Bhavsar AP, Visscher H, Rassekh SR, Li Y, Lee JW, et al. A coding variant in RARG confers susceptibility to anthracycline-induced cardiotoxicity in childhood cancer. Nature Genetics. 2015;47(9):1079-84.

60. Velensek V, Mazic U, Krzisnik C, Demsar D, Jazbec J, Jereb B. Cardiac damage after treatment of childhood cancer: A long-term follow-up. BMC Cancer. 2008;8 (no pagination)(141).

61. Landy DC, Miller TL, Lipsitz SR, Lopez-Mitnik G, Hinkle AS, Constine LS, et al. Cranial irradiation as an additional risk factor for anthracycline cardiotoxicity in childhood cancer survivors: An analysis from the cardiac risk factors in childhood cancer survivors study. Pediatric Cardiology. 2013;34(4):826-34.

62. Sorensen K, Levitt GA, Bull C, Dorup I, Sullivan ID. Late anthracycline cardiotoxicity after childhood cancer: A prospective longitudinal study. Cancer. 2003;97(8):1991-8.

63. Nolan MT, Marwick TH, Plana JC, Li Z, Ness KK, Joshi VM, et al. Effect of Traditional Heart Failure Risk Factors on Myocardial Dysfunction in Adult Survivors of Childhood Cancer. JACC: Cardiovascular Imaging. 2018;11(8):1202-3.

64. Mulrooney DA, Armstrong GT, Huang S, Ness KK, Ehrhardt MJ, Joshi VM, et al. Cardiac outcomes in adult survivors of childhood cancer exposed to cardiotoxic therapy. Annals of Internal Medicine. 2016;164(2):93-101.

65. Spewak MB, Williamson RS, Mertens AC, Border WL, Meacham LR, Wasilewski-Masker KJ. Yield of screening echocardiograms during pediatric follow-up in survivors treated with anthracyclines and cardiotoxic radiation. Pediatric Blood and Cancer. 2017;64 (6) (no pagination)(e26367).

66. Ramjaun A, Alduhaiby E, Ahmed S, Wang L, Yu E, Nathan PC, et al. Echocardiographic Detection of Cardiac Dysfunction in Childhood Cancer Survivors: How Long Is Screening Required? Pediatric Blood and Cancer. 2015;62(12):2197-203.

67. Christiansen JR, Kanellopoulos A, Lund MB, Massey R, Dalen H, Kiserud CE, et al. Impaired exercise capacity and left ventricular function in long-term adult survivors of childhood acute lymphoblastic leukemia. Pediatric Blood and Cancer. 2015;62(8):1437-43.

68. Christiansen JR, Massey R, Dalen H, Kanellopoulos A, Hamre H, Ruud E, et al. Right ventricular function in long-term adult survivors of childhood lymphoma and acute lymphoblastic leukaemia. Eur Heart J Cardiovasc Imaging. 2016;17(7):735-41.

69. Abaza A, El-Shanshoury H. Risk assessment of radio-chemotherapy in pediatric soft tissue sarcomas. Journal of Radiation Research and Applied Sciences. 2015;8(1):110-9.

70. Kovacs GT, Erlaky H, Toth K, Horvath E, Szabolcs J, Csoka M, et al. Subacute cardiotoxicity caused by anthracycline therapy in children: Can dexrazoxane prevent this effect? European Journal of Pediatrics. 2007;166(11):1187-8.

71. Conyers R, Costello B, La Gerche A, Tripaydonis A, Burns C, Ludlow L, et al. Chemotherapy-related cardiotoxicity: are Australian practitioners missing the point? Internal Medicine Journal. 2017;47(10):1166-72.

72. Hu H, Zhang W, Huang D, Yang Q, Li J, Gao Y. Cardiotoxicity of anthracycline (ANT) treatment in children with malignant tumors. Pediatric Hematology and Oncology. 2018;35(2):111-20.

73. Elbl L, Hrstkova H, Tomaskova I, Michalek J. Late anthracycline cardiotoxicity protection by dexrazoxane (ICRF-187) in pediatric patients: Echocardiographic follow-up. Supportive Care in Cancer. 2006;14(2):128-36.

74. Iarussi D, Galderisi M, Ratti G, Tedesco MA, Indolfi P, Casale F, et al. Left ventricular systolic and diastolic function after anthracycline chemotherapy in childhood. Clinical Cardiology. 2001;24(10):663-9.

75. Iarussi D, Indolfi P, Pisacane C, Casale F, Martino V, Fusco A, et al. Comparison of left ventricular function by echocardiogram in patients with Wilms' tumor treated with anthracyclines versus those not so treated. American Journal of Cardiology. 2003;92(3):359-61.

76. Juergens C, Weston C, Lewis I, Whelan J, Paulussen M, Oberlin O, et al. Safety assessment of intensive induction with vincristine, ifosfamide, doxorubicin, and etoposide (VIDE) in the treatment of ewing tumors in the EURO-E.W.I.N.G. 99 Clinical Trial. Pediatric Blood and Cancer. 2006;47(1):22-9.

77. Kupeli S. Evaluation of coronary artery disease by computed tomography angiography in patients treated for Hodgkin lymphoma. Asia Pacific Journal of Oncology and Hematology. 2010;2(2).

78. Langer T, Stohr W, Bielack S, Paulussen M, Treuner J, Beck JD. Late Effects Surveillance System for Sarcoma Patients. Pediatric Blood and Cancer. 2004;42(4):373-9.

79. Lipshultz SE, Giantris AL, Lipsitz SR, Dalton VK, Asselin BL, Barr RD, et al. Doxorubicin administration by continuous infusion is not cardioprotective: The Dana-Farber 91-01 acute lymphoblastic leukemia protocol. Journal of Clinical Oncology. 2002;20(6):1677-82.

80. Lipshultz SE, Lipsitz SR, Kutok JL, Miller TL, Colan SD, Neuberg DS, et al. Impact of hemochromatosis gene mutations on cardiac status in doxorubicin-treated survivors of childhood high-risk leukemia. Cancer. 2013;119(19):3555-62.

81. Lipshultz SE, Miller TL, Lipsitz SR, Neuberg DS, Dahlberg SE, Colan SD, et al. Continuous versus bolus infusion of doxorubicin in children with ALL: Long-term cardiac outcomes. Pediatrics. 2012;130(6):1003-11.

82. Lipshultz SE, Scully RE, Lipsitz SR, Sallan SE, Silverman LB, Miller TL, et al. Assessment of dexrazoxane as a cardioprotectant in doxorubicin-treated children with high-risk acute lymphoblastic leukaemia: Long-term follow-up of a prospective, randomised, multicentre trial. The Lancet Oncology. 2010;11(10):950-61.

83. Mohta R, Saxena A, Jain Y, Gupta S, Thavaraj V, Narain S, et al. Anthracycline associated cardiac toxicity in children with malignancies. Indian Pediatrics. 2002;39(6):549-55.

84. Moussa E, Zamzam M, Kamel A, Salah Z, Attia I, Gaber L, et al. Risk stratification and pattern of cardiotoxicity in pediatric Ewing sarcoma. Journal of the Egyptian National Cancer Institute. 2017;29(1):53-6.

85. Oztarhan K, Guler S, Aktas B, Arslan M, Salcioglu Z, Aydogan G. The value of echocardiography versus cardiac troponin i levels in the early detection of anthracycline cardiotoxicity in childhood acute leukemia: Prospective evaluation of a 7-year-long clinical follow-up. Pediatric Hematology and Oncology. 2011;28(5):380-94.

86. Pejcic L, Vasic K. Preliminary testing of anthracycline-induced cardiotoxicity in children. Journal of BUON. 2017;22(6):1611-2.

87. Shiroya-Wandabwa M, Yuko-Jowi C, Nduati R, Githanga J, Wamalwa D. Risk factors for cardiac dysfunction in children on treatment for cancer at Kenyatta National Hospital, Nairobi. East African medical journal. 2009;86(12 Suppl):S52-7.

88. Smibert E, Carlin JB, Vidmar S, Wilkinson LC, Newton M, Weintraub RG. Exercise echoicardiography reflects cumulative anthracycline exposure during childhood. Pediatric Blood and Cancer. 2004;42(7):556-62.

89. Peeters J, Meitert J, Paulides M, Beck JD, Langer T. Late effects surveillance system after childhood cancer in Germany, austria and parts of Switzerland--update 2009. Strahlentherapie und Onkologie : Organ der Deutschen Rontgengesellschaft [et al]. 2009;185 Suppl 2:5-7.

90. Stapleton GE, Stapleton SL, Martinez A, Ayres NA, Kovalchin JP, Bezold LI, et al. Evaluation of Longitudinal Ventricular Function with Tissue Doppler Echocardiography in Children Treated with Anthracyclines. Journal of the American Society of Echocardiography. 2007;20(5):492-7.

91. Temming P, Qureshi A, Hardt J, Leiper AD, Levitt G, Ancliff PJ, et al. Prevalence and predictors of anthracycline cardiotoxicity in children treated for acute myeloid leukaemia: Retrospective cohort study in a single centre in the United Kingdom. Pediatric Blood and Cancer. 2011;56(4):625-30.

92. Tran JC, Ruble K, Loeb DM, Chen AR, Thompson WR. Automated Functional Imaging by 2D Speckle Tracking Echocardiography Reveals High Incidence of Abnormal Longitudinal Strain in a Cohort of Pediatric Oncology Patients. Pediatric Blood and Cancer. 2016.

93. Uderzo C, Pillon M, Corti P, Tridello G, Tana F, Zintl F, et al. Impact of cumulative anthracycline dose, preparative regimen and chronic graft-versus-host disease on pulmonary and cardiac function in children 5 years after allogeneic hematopoietic stem cell transplantation: A prospective evaluation on behalf of the EBMT Pediatric Diseases and Late Effects Working Parties. Bone Marrow Transplantation. 2007;39(11):667-75.

94. Wang H, Chi ZF, Li S, Wang XL, Hao LC. [Efficacy, side effects and blood concentration monitoring of high-dose methotrexate in treatment of 180 children with acute lymphoblastic leukemia]. Zhongguo shi yan xue ye xue za zhi. 2011;19(4):949-52.

95. Yu W, Tang L, Lin F, Yao Y, Shen Z. Pirarubicin versus doxorubicin in neoadjuvant/adjuvant chemotherapy for stage IIB limb high-grade osteosarcoma: does the analog matter? Medical oncology (Northwood, London, England). 2015;32(1):307.

96. Markman TM, Ruble K, Loeb D, Chen A, Zhang Y, Beasley GS, et al. Electrophysiological effects of anthracyclines in adult survivors of pediatric malignancy. Pediatric Blood and Cancer. 2017;64 (11) (no pagination)(e26556).

97. Abosoudah I, Greenberg ML, Ness KK, Benson L, Nathan PC. Echocardiographic surveillance for asymptomatic late-onset anthracycline cardiomyopathy in childhood cancer survivors. Pediatric Blood and Cancer. 2011;57(3):467-72.

98. Creutzig U, Diekamp S, Zimmermann M, Reinhardt D. Longitudinal evaluation of early and late anthracycline cardiotoxicity in children with AML. Pediatric Blood and Cancer. 2007;48(7):651-62.

99. Elbl L, Hrstkova H, Chaloupka V. The late consequences of anthracycline treatment on left ventricular function after treatment for childhood cancer. European Journal of Pediatrics. 2003;162(10):690-6.

100. Marx M, Langer T, Graf N, Hausdorf G, Stohr W, Ludwig R, et al. Multicentre analysis of anthracycline-induced cardiotoxicity in children following treatment according to the nephroblastoma studies SIOP No.9/GPOH and SIOP 93-01/GPOH. Medical and Pediatric Oncology. 2002;39(1):18-24.

101. Hori H, Kudoh T, Nishimura S, Oda M, Yoshida M, Hara J, et al. Acute and late toxicities of pirarubicin in the treatment of childhood acute lymphoblastic leukemia: results from a clinical trial by the Japan Association of Childhood Leukemia Study. International Journal of Clinical Oncology. 2017;22(2):387-96.

102. Chen C, Heusch A, Donner B, Janssen G, Gobel U, Schmidt KG. Present risk of anthracycline or radiation-induced cardiac sequelae following therapy of malignancies in children and adolescents. Klinische Padiatrie. 2009;221(3):162-6.

103. Barlogis V, Auquier P, Bertrand Y, Chastagner P, Plantaz D, Poiree M, et al. Late cardiomyopathy in childhood acute myeloid leukemia survivors: A study from the L.E.A. program. Haematologica. 2015;100(5):e186-e9.

104. Andolina JR, Dilley K. Anthracycline-induced cardiac toxicity more likely in underweight childhood cancer survivors. Journal of Pediatric Hematology/Oncology. 2010;32(5):411-5.

105. Sagi JC, Egyed B, Kelemen A, Kutszegi N, Hegyi M, Gezsi A, et al. Possible roles of genetic variations in chemotherapy related cardiotoxicity in pediatric acute lymphoblastic leukemia and osteosarcoma. BMC Cancer. 2018;18 (1) (no pagination)(704).

106. Cox CL, Rai SN, Rosenthal D, Phipps S, Hudson MM. Subclinical late cardiac toxicity in childhood cancer survivors: Impact on self-reported health. Cancer. 2008;112(8):1835-44.

107. Cox CL, Rai SN, Rosenthal D, Phipps S, Hudson MM. Subclinical late cardiotoxicity and its effect on self-reported health for survivors of childhood cancer. American Journal of Hematology/ Oncology. 2008;7(11).

108. Lipshultz SE, Landy DC, Lopez-Mitnik G, Lipsitz SR, Hinkle AS, Constine LS, et al. Cardiovascular status of childhood cancer survivors exposed and unexposed to cardiotoxic therapy. Journal of Clinical Oncology. 2012;30(10):1050-7.

109. Desai L, Balmert L, Reichek J, Hauck A, Gambetta K, Webster G. Electrocardiograms for cardiomyopathy risk stratification in children with anthracycline exposure. Cardio-Oncology. 2019;5(1).

110. Armstrong GT, Plana JC, Zhang N, Srivastava D, Green DM, Ness KK, et al. Screening adult survivors of childhood cancer for cardiomyopathy: Comparison of echocardiography and cardiac magnetic resonance imaging. Journal of Clinical Oncology. 2012;30(23):2876-84.

111. Aznar EGC, Casas AA, Escribano MACC, Montanes LJ, Aizpun JIL, Villagrasa PS. Echocardiographic evolution of left ventricular function in childhood leukemia survivors. Current Problems in Cancer. 2018;42(4):397-408.

112. Eidem BW, Sapp BG, Suarez CR, Cetta F. Usefulness of the myocardial performance index for early detection of Anthracycline-induced cardiotoxicity in children. American Journal of Cardiology. 2001;87(9):1120-2.

113. Gonzalez Otero A, Machin Garcia S, Arencibia Nunez A, Collazo Acosta J, Rivera Keeling C, Bravo Perez De Ordaz L, et al. Longitudinal ecocardiographic assesment in patients with acute lymphoid leukemia that received anthracyclines during childhood. [Spanish]. Revista Cubana de Hematologia, Inmunologia y Hemoterapia. 2016;32(4):470-82.

114. Jarfelt M, Andersen NH, Glosli H, Jahnukainen K, Jonmundsson GK, Malmros J, et al. Cardiac function in survivors of childhood acute myeloid leukemia treated with chemotherapy only: a NOPHO-AML study. European Journal of Haematology. 2016;97(1):55-62.

115. Myrdal OH, Kanellopoulos A, Christensen JR, Ruud E, Edvardsen E, Kongerud J, et al. Risk factors for impaired pulmonary function and cardiorespiratory fitness in very long-term adult survivors of childhood acute lymphoblastic leukemia after treatment with chemotherapy only<sup>*</sup>. Acta Oncol. 2018;57(5):658-64.

116. Rotz SJ, Powell A, Myers KC, Taylor MD, Jefferies JL, Lane A, et al. Treatment exposures stratify need for echocardiographic screening in asymptomatic long-term survivors of hematopoietic stem cell transplantation. Cardiol Young. 2019:1-6.

117. Seth R, Singh A, Seth S, Sapra S. Late effects of treatment in survivors of childhood cancers: A single-centre experience. Indian Journal of Medical Research. 2017;146(August):216-23.

118. Sieswerda E, Kremer LCM, Vidmar S, De Bruin ML, Smibert E, Sjoberg G, et al. Exercise echocardiography in asymptomatic survivors of childhood cancer treated with anthracyclines: A prospective follow-up study. Pediatric Blood and Cancer. 2010;54(4):579-84.

119. AbdelHameid D, Mills A, Dean J, Piguet N, Shankar S. Long term effects of therapy among childhood cancer survivors treated in the last two decades. Pediatric Hematology Oncology Journal. 2019;4(1):12-6.

120. Georgakopoulos P, Kyriakidis M, Perpinia A, Karavidas A, Zimeras S, Mamalis N, et al. The Role of Metoprolol and Enalapril in the Prevention of Doxorubicin-induced Cardiotoxicity in Lymphoma Patients. Anticancer research. 2019;39(10):5703-7.

121. Tong X, Li VWY, Liu APY, So EKF, Chan Q, Ho KKH, et al. Cardiac Magnetic Resonance T1 Mapping in Adolescent and Young Adult Survivors of Childhood Cancers. Circulation. 2019;Cardiovascular imaging. 12(4):e008453.

122. Morales JS, Santana-Sosa E, Santos-Lozano A, Bano-Rodrigo A, Valenzuela PL, Rincon-Castanedo C, et al. Inhospital exercise benefits in childhood cancer: A prospective cohort study. Scandinavian journal of medicine & science in sports. 2020;30(1):126-34.

123. Creutzig U, Zimmermann M, Bourquin JP, Dworzak MN, Fleischhack G, Graf N, et al. Randomized trial comparing liposomal daunorubicin with idarubicin as induction for pediatric acute myeloid leukemia: Results from study AML-BFM 2004. Blood. 2013;122(1):37-43.

124. Allen J, Thomson JDR, Lewis IJ, Gibbs JL. Mitral regurgitation after anthracycline treatment for childhood malignancy. Heart. 2001;85(4):430-2.

125. Armendariz H, Fernandez Barbieri MA, Freigeiro D, Lastiri F, Felice MS, Dibar E. Treatment strategy and long-term results in pediatric patients treated in two consecutive AML-GATLA trials [1]. Leukemia. 2005;19(12):2139-42.

126. Bhakta N, Liu Q, Yeo F, Baassiri M, Ehrhardt MJ, Srivastava DK, et al. Cumulative burden of cardiovascular morbidity in paediatric, adolescent, and young adult survivors of Hodgkin's lymphoma: an analysis from the St Jude Lifetime Cohort Study. The Lancet Oncology. 2016;17(9):1325-34.

127. Dorup I, Levitt G, Sullivan I, Sorensen K. Prospective longitudinal assessment of late anthracycline cardiotoxicity after childhood cancer: The role of diastolic function. Heart. 2004;90(10):1214-6.

128. Ginsberg JP, Cnaan A, Zhao H, Clark BJ, Paridon SM, Chin AJ, et al. Using health-related quality of life measures to predict cardiac function in survivors exposed to anthracyclines. Journal of Clinical Oncology. 2004;22(15):3149-55.

129. Rigon H, Lopes LF, Latorre MDR, De Camargo B. The GEPETTO program for surveillance of long-term survivors of childhood cancer: Preliminary report from a single institution in Brazil. Medical and Pediatric Oncology. 2003;40(6):405-6.

130. Van Dijk IWEM, Oldenburger F, Cardous-Ubbink MC, Geenen MM, Heinen RC, De Kraker J, et al. Evaluation of late adverse events in long-term Wilms' tumor survivors. International Journal of Radiation Oncology Biology Physics. 2010;78(2):370-8.

131. Wong FL, Bhatia S, Landier W, Francisco L, Leisenring W, Hudson MM, et al. Cost-effectiveness of the children's oncology group long-term follow-up screening guidelines for childhood cancer survivors at risk for treatment-related heart failure. Annals of Internal Medicine. 2014;160(10):672-83.

132. Yeh JM, Nohria A, Diller L. Routine echocardiography screening for asymptomatic left ventricular dysfunction in childhood cancer survivors: A model-based estimation of the clinical and economic effects. Annals of Internal Medicine. 2014;160(10):661-71.

133. Galper SL, Yu JB, Mauch PM, Strasser JF, Silver B, Lacasce A, et al. Clinically significant cardiac disease in patients with Hodgkin lymphoma treated with mediastinal irradiation. Blood. 2011;117(2):412-8.

134. Lipshultz SE, Lipsitz SR, Sallan SE, Dalton VM, Mone SM, Gelber RD, et al. Chronic progressive cardiac dysfunction years after doxorubicin therapy for childhood acute lymphoblastic leukemia. Journal of clinical oncology : official journal of the American Society of Clinical Oncology. 2005;23(12):2629-36.

135. Cheung YF, Yu W, Cheuk DKL, Cheng FWT, Yang JYK, Yau JPW, et al. Plasma High Sensitivity Troponin T Levels in Adult Survivors of Childhood Leukaemias: Determinants and Associations with Cardiac Function. PLoS ONE. 2013;8 (10) (no pagination)(e77063).

136. Landier W, Armenian SH, Lee J, Thomas O, Wong FL, Francisco L, et al. Yield of Screening for Long-Term Complications Using the Children's Oncology Group Long-Term Follow-Up Guidelines. Journal of Clinical Oncology. 2012;30(35):4401-8.

137. Bates JE, Howell RM, Liu Q, Yasui Y, Mulrooney DA, Dhakal S, et al. Therapy-related cardiac risk in childhood cancer survivors: An analysis of the childhood cancer survivor study. Journal of Clinical Oncology. 2019;37(13):1090-101.

138. Khanna A, Pequeno P, Gupta S, Thavendiranathan P, Lee DS, Abdel-Qadir H, et al. Increased Risk of All Cardiovascular Disease Subtypes among Childhood Cancer Survivors: Population-Based Matched Cohort Study. Circulation. 2019;140(12):1041-3.

139. Zaucha-Prazmo A, Kowalczyk JR, Sadurska E. Early evaluation of the cardiovascular system in children treated for neoplastic diseases with dexrazoxane prophylaxis. [Polish]. Pediatria Polska. 2003;78(5):385-9.

140. Krajinovic M, Elbared J, Drouin S, Bertout L, Rezgui A, Ansari M, et al. Polymorphisms of ABCC5 and NOS3 genes influence doxorubicin cardiotoxicity in survivors of childhood acute lymphoblastic leukemia. Pharmacogenomics J. 2017;17(1):107.

141. Gershanovich ML. Cardioxan: prevention of anthracycline-related cardiotoxicity. [Russian]. Voprosy onkologii. 2004;50(4):482-91.

142. Erlaky H, Toth K, Szabolcs J, Horvath E, Kemeny V, Muller J, et al. Subacute cardiotoxicity caused by anthracycline therapy in children: can dexrazoxane prevent this effect?. [Hungarian]. Magyar onkologia. 2006;50(1):25-32.

143. Armenian SH, Gelehrter SK, Vase T, Venkatramani R, Lier W, Wilson KD, et al. Screening for cardiac dysfunction in anthracycline-exposed childhood cancer survivors. Clinical Cancer Research. 2014;20(24):6314-23.

144. Mulrooney DA, Armstrong GT, Huang S, Ness KK, Ehrhardt MJ, Joshi VM, et al. Cardiac Outcomes in Adult Survivors of Childhood Cancer Exposed to Cardiotoxic Therapy: A Cross-sectional Study. Ann Intern Med. 2016;164(2):93-101.

145. Semsei AF, Erdelyi DJ, Ungvari I, Csagoly E, Hegyi MZ, Kiszel PS, et al. ABCC1 polymorphisms in anthracycline-induced cardiotoxicity in childhood acute lymphoblastic leukaemia. Cell Biology International. 2012;36(1):79-86.

146. Guldner L, Haddy N, Pein F, Diallo I, Shamsaldin A, Dahan M, et al. Radiation dose and long term risk of cardiac pathology following radiotherapy and anthracyclin for a childhood cancer. Radiotherapy and Oncology. 2006;81(1):47-56.

147. Armenian SH, Gelehrter SK, Vase T, Venkatramani R, Landier W, Wilson KD, et al. Carnitine and cardiac dysfunction in childhood cancer survivors treated with anthracyclines. Cancer Epidemiology Biomarkers and Prevention. 2014;23(6):1109-14.

148. Mavinkurve-Groothuis AMC, Groot-Loonen J, Bellersen L, Pourier MS, Feuth T, Bokkerink JPM, et al. Abnormal nt-pro-bnp levels in asymptomatic long-term survivors of childhood cancer treated with anthracyclines. Pediatric Blood and Cancer. 2009;52(5):631-6.

149. Hudson MM, Ness KK, Gurney JG, Mulrooney DA, Chemaitilly W, Krull KR, et al. Clinical ascertainment of health outcomes among adults treated for childhood cancer. JAMA. 2013;309(22):2371-81.

150. Elbl L, Hrstkova H, Chaloupka V, Michalek J. Dynamic stress echocardiography in asymptomatic patients who received chemotherapy in childhood because of a malignant disease. Kardiologia Polska. 2003;58(3):190-6.

151. Ness KK, Plana JC, Joshi VM, Luepker RV, Durand JB, Green DM, et al. Exercise intolerance, mortality, and organ system impairment in adult survivors of childhood cancer. Journal of Clinical Oncology. 2020;38(1):29-42.

152. Brouwer CAJ, Postma A, Vonk JM, Zwart N, Van Den Berg MP, Bink-Boelkens MTE, et al. Systolic and diastolic dysfunction in long-term adult survivors of childhood cancer. European Journal of Cancer. 2011;47(16):2453-62.
